# Binary Hydrogels: Induction Methods and Recent Application Progress as Food Matrices for Bioactive Compounds Delivery—A Bibliometric Review

**DOI:** 10.3390/gels9010068

**Published:** 2023-01-14

**Authors:** Adonis Hilal, Anna Florowska, Małgorzata Wroniak

**Affiliations:** Department of Food Technology and Assessment, Institute of Food Science, Warsaw University of Life Sciences, 02-787 Warsaw, Poland

**Keywords:** proteins, polysaccharides, hydrogels, functional properties, delivery systems, bioactive ingredients, plant-based food, food development

## Abstract

Food hydrogels are biopolymeric materials made from food-grade biopolymers with gelling properties (proteins and polysaccharides) and a 3D network capable of incorporating large amounts of water. They have sparked considerable interest because of their potential and broad application range in the biomedical and pharmaceutical sectors. However, hydrogel research in the field of food science is still limited. This knowledge gap provides numerous opportunities for implementing their unique properties, such as high water-holding capacity, moderated texture, compatibility with other substances, cell biocompatibility, biodegradability, and high resemblance to living tissues, for the development of novel, functional food matrices. For that reason, this article includes a bibliometric analysis characterizing research trends in food protein–polysaccharide hydrogels (over the last ten years). Additionally, it characterizes the most recent developments in hydrogel induction methods and the most recent application progress of hydrogels as food matrices as carriers for the targeted delivery of bioactive compounds. Finally, this article provides a future perspective on the need to evaluate the feasibility of using plant-based proteins and polysaccharides to develop food matrices that protect nutrients, including bioactive substances, throughout processing, storage, and digestion until they reach the specific targeted area of the digestive system.

## 1. Introduction

Hydrogels are viscoelastic aqueous matrices composed of crosslinked polymer chains forming a three-dimensional hydrophilic network. This three-dimensional system contains molecules, fibers, or particles, with water or an aqueous phase serving as the dispersion medium [1]. The hydrophilic character of hydrogels is caused by some hydrophilic residues (such as amino, carboxyl, and hydroxyl groups) of the polymer(s), along with the nature and density of the formed network connections. Such networks can hold large amounts of water (even 99% *w*/*w*) in their structure while maintaining solid-like properties [2]. The type (physical or chemical) and density (number of crosslinks) of network connections formed by these polymers help to maintain the final gel network. As a result, the structure, viscoelasticity, and water-holding capacity of hydrogels are highly dependent on the polymer source (natural or synthetic), method of preparation (induction method), ionic charge, and the size of the network [3].

Hydrogel materials are widely used, with significant applications in medical, cosmetics, textiles, agriculture, and recently in the food sector as well. Because of their broad range of applicational potential, researchers have been studying hydrogels for years. The biomedical and pharmaceutical industries have primarily implemented hydrogels as delivery systems [4,5], scaffolds for cell cultivation [6], and tissue engineering [7]. However, when it comes to the food industry, the implementation of hydrogels is constrained by restrictions on the use of certain ingredients that need to be food-grade, generally recognized as safe (GRAS) by the Food and Drug Administration (FDA) in the USA and included in the EU list of permitted food additives laid down in Regulation EC 1333/2008. According to their origin, food-grade biopolymers are divided into proteins and polysaccharides. These biopolymers have a great potential to address today’s consumer health and environmental sustainability concerns since they are renewable, affordable, biocompatible, biodegradable, and edible, as well as having a wide range of functionalities and gelation routes [8]. Proteins and polysaccharides are primary functional components in developing food colloidal systems since they can create and modify food matrix structures, textures, sensory properties, and shelf life.

Protein-based hydrogels are formed when the protein molecules unfold, revealing hydrophilic and thiol groups [9]. This unfolding of the structure (denaturation) process can be initiated by heating, pH, or salt modulation. The denaturation allows the chains to interact via covalent interactions (by hydrophobic or electrostatic interactions, hydrogen bond formation, and less frequently, disulfide bond formation). The covalent interaction between the polymer chains leads to their aggregation, forming a three-dimensional gel structure [10]. Among plant-based globular proteins, such as soy [11,12], pea [10,13], wheat [14,15], and zein [16,17] are reported in the literature to have an excellent gelling ability, similar to their animal-based proteins counterparts (whey and egg) [18,19]. The formation of polysaccharide hydrogels is less complicated than that of globular proteins. Polysaccharide hydrogel formation can be induced through various methods, among others heat and cooling, pH and salt modulation, the addition of sucrose, and freeze-thaw cycles [20]. Among the widely used in the food industry polysaccharides that have gelling abilities are carrageenan [21,22], chitosan [23,24], alginate [25,26], inulin [27,28], starch [29,30], cellulose [31,32], gum arabic [33,34], gellan gum [35,36], etc.

Binary hydrogels composed of proteins and polysaccharides were developed to avoid some of the limitations such as poor water holding capacity and weak gel strength, physical instability, etc. imposed by hydrogels prepared with a single biopolymer [37]. A different combination of proteins and polysaccharides can be used to create such binary hydrogels: protein–protein, polysaccharide–polysaccharide, and protein–polysaccharide [38]. Proteins and polysaccharides can effectively form binary hydrogels due to their ability to interact with each other via non-covalent and covalent interactions [39]. Furthermore, when the concentration of one biopolymer is insufficient to form a stable hydrogel, adding another biopolymer as a filler component can improve the physicochemical properties of the system, allowing the formation of a network structure [40,41]. A wide range of protein–polysaccharide binary hydrogels with various microstructures and physicochemical properties can be obtained based on the interaction between those two biopolymers, the individual properties of each used component, and the applied induction conditions [42]. An example would be a binary hydrogel composed of a whey protein/starch mixture, distinguished by new and intriguing properties [43]. It was discovered that the synergistic interactions between casein and carrageenan also improved hydrogel’s rheological and microstructural properties [44]. Zernov et al. [45] reported that mixing chitosan and collagen makes it possible to produce a hydrogel that can act as an edible microcarrier for cultured meat. Furthermore, soy protein—a model plant-based protein mixed with polysaccharides—can form binary hydrogel and gain new properties as a food ingredient [46,47]. Combinations of soy protein gels and polysaccharides tested by other researchers are as follows: soy protein–sodium alginate hydrogel [48], soy protein–carrageenan hydrogel [49], soy protein–inulin hydrogel [50], soy protein–corn fiber gum hydrogel [51]. Other plant-based proteins and polysaccharides are also being studied regarding their ability to form binary hydrogels. Among them, the most popular in the literature are pea protein–sodium alginate hydrogel [52], pea protein–soluble soybean polysaccharide hydrogel [53], and zein protein–pectin hydrogel [54].

Since studies on the topic of food hydrogels are still minimal in comparison to biomedical or pharmaceutical hydrogels, there are immense opportunities to contribute to the development of the food industry through a cross-integration between areas with advanced knowledge. Regarding model behavior, food biopolymer hydrogels might be more complex than synthetic hydrogels [37]. Despite this, a proper hydrogel design based on a thorough understanding of the mechanisms in food matrices can improve the final food matrix’s quality, nutrition, and nutrient bioavailability [41,55]. Therefore, using a bibliometric analysis as a research performance investigation tool for detailed databases can reveal trends and patterns in scientific research areas worldwide. This statistical tool has raised researchers’ considerable interest in providing an in-depth view of the advancements in binary hydrogels’ food processing and applications [41,56,57]. The purpose of conducting a bibliometric analysis is not to discuss the findings of the identified papers but to characterize research trends in a chosen field of knowledge [58,59]. The significance of this manuscript is to provide a mixed review that combines bibliometric analysis and a literature review of the latest developments in hydrogel induction methods and the present research findings on the topic of protein–polysaccharide hydrogels as a food matrix.

## 2. Methodological Procedures

The article presents a literature review emphasizing protein–polysaccharide hydrogel induction methods and the application progress of protein–polysaccharide hydrogels as food matrices to supplement the information provided by the bibliometric analysis.

In this study, a mixed methodology was carried out, including a bibliometric analysis of papers obtained from the Scopus database (https://www.scopus.com/search/form.uri?display=advanced, accessed on 1 December 2022) and a literature review emphasizing the induction methods and the application progress of protein–polysaccharide hydrogels as food matrices. A survey was carried out in the Scopus database (in October 2022) to access the papers used to perform the bibliometric analysis. The methodological procedure adopted for the bibliometric analysis was divided into two general phases, the data collection phase, and the data mapping/visualization phase.

The entered query string included the terms “protein polysaccharide food hydrogels”, “food biopolymer hydrogel”, and “food hydrogel” as search words in the publication’s titles and abstracts. The publication timeframe was set from 2012 to 2022, and the types of documents were considered: articles and reviews. Some words were excluded (e.g., aerogels, oleogels, male, female), as well as some research areas (e.g., economics and finance, computer science, business management and accounting, mathematics, social sciences, energy, planetary sciences, neuroscience, nursing, and health professions) to refine the study. A result of 297 documents was obtained, of which 239 were articles and 58 were reviews, all in the final publication stage.

The data mapping/visualization phase was accomplished using a state-of-art scientometric mapping tool provided by VOSviewer software (version 1.6.18, CWTS, Leiden, The Netherlands). The data, including all the details regarding the 297 documents found by the search engine in the Scopus database, were exported, and a performance analysis was carried out to discover the general patterns of research on protein–polysaccharide hydrogels. A cluster analysis was carried out based on the keywords co-occurrence and the bibliographic coupling of in-country collaborations [60,61,62,63,64].

## 3. Bibliometric Analysis

**Figure 1:**
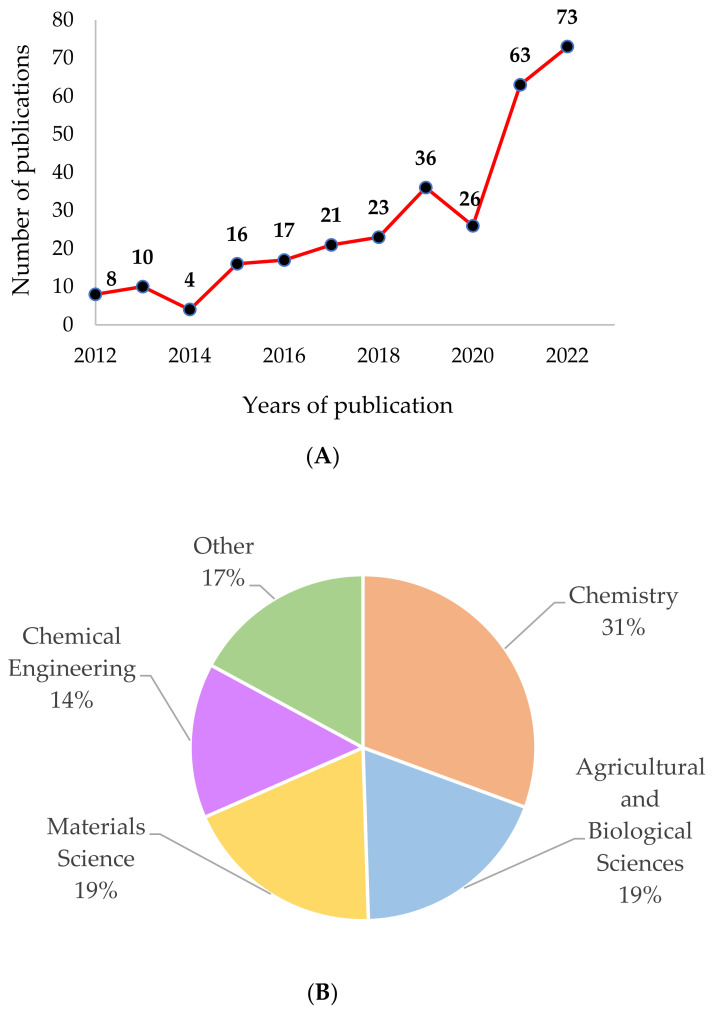
Evaluation of the scientific publications. (**A**) The number of publications registered on the topic of protein–polysaccharide food hydrogels over the last ten years; (**B**) the main subject area in which the publications registered on the topic of protein–polysaccharide food hydrogels over the last ten years (research carried out in the Scopus database in October 2022).

A total of 297 documents were analyzed, of which 80.5% were articles and 19.5% were review papers. Figure 1 shows the evaluation of the scientific publication on protein-polysaccharide food hydrogels registered in the Scopus database in 2012–2022. Figure 1A shows the number of publications, and Figure 1B represents the main subject area of the publications.

By analyzing the data presented in Figure 1A, a slow but systematic growth of the number of publications, it in years 2014–2019, can be observed. Currently, since 2020, there has been a dynamic increase in the number of published articles on protein–polysaccharide food hydrogels. In 2020, the number of publications on this topic reached 63, and in 2022—73. The growth in the number of published documents reflects the awareness of the potential uses of hydrogels in the food sector. This growth could be caused by the food industry’s growing concern about providing enough nutritious food for everyone while protecting natural resources. This growing concern has resulted in the faster development of plant-based foods and hybrid food products (from animal and plant sources), which have emerged as a new growing trend that can help the sustainability challenge [65]. The growing interest in the development of plant-based foods (including hybrid foods) has increased the number of studies on food hydrogels, which have the potential to improve the appearance, texture, flavor, mouthfeel, and functionality of these new products [20,37,66,67,68].

The scientific papers that addressed the topic of protein–polysaccharide food hydrogels were published mainly in four subject areas (Figure 1B): chemistry (31% of published documents), agriculture and biological sciences (19%, which include food science), materials science (19%), and chemical engineering area (14%). The other areas in which the analyzed scientific documents were published were physics and astronomy (7.7%); biochemistry, genetics, and molecular biology (5.9%); pharmacology, toxicology, and pharmaceutics (1.6%); immunology and microbiology (1.5%); medicine (0.5%). These research areas prove the interdisciplinary aspect of protein–polysaccharide food hydrogels [1,8].

**Figure 2:**
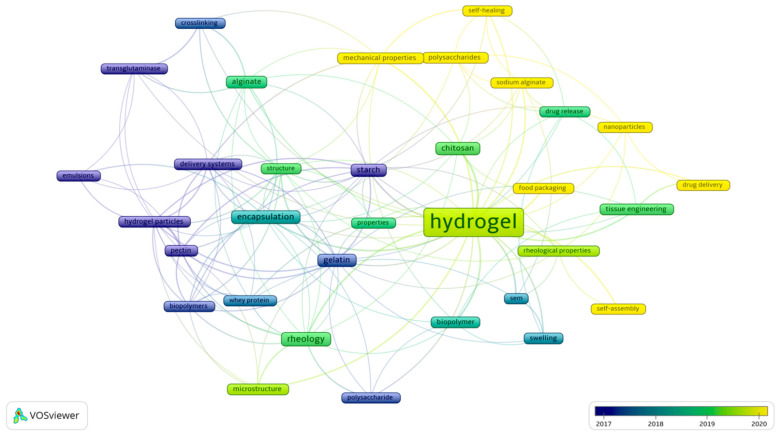
Visualization of the keywords network based on their co-occurrence. The frame size represents the frequency of the keyword’s co-occurrence. The color scale represents the average number of document publications per year.

Citation is one of the most critical indicators of a publication’s relevance [69]. Table 1 provides the most cited publications during the ten years. Moreover, to determine the current trends in scientific research on the topic of protein–polysaccharide food hydrogels, the keywords’ co-occurrence in the studied documents was performed. It can be observed that the most recent publications are related to the topic of polysaccharide hydrogels and hydrogels properties, such as self-healing, self-assembly, and mechanical properties (Figure 2).

Most of the studies concerned the application of hydrogels in food packaging and drug delivery. Auriemma et al. [79] stated that polysaccharide hydrogels have a promising potential in developing drug delivery systems aimed at controlling and targeting the delivery of many drugs. Despite their potential, many breakthroughs in clinical studies of the release mechanisms are needed to use these hydrogels as drug carriers while also focusing on the SbD (safe-by-design) approach. The standardization of the analysis regarding the release mechanisms of hydrogel delivery systems is a crucial topic in the meaningful, intelligent delivery systems design [80,81]. Protein–polysaccharide food hydrogels have received significant attention because of the growing need to replace plastic packaging with new, safe, and biodegradable materials. Additionally, researchers are trying to implement the knowledge from disciplines, such as the pharmaceutical one, to develop hydrogel-based packaging materials with the ability to release bioactive compounds that could prevent the growth of harmful microorganisms while protecting the food product from moisture and nutrient loss [82,83]. The study of co-occurring keywords helped isolate two main interlinked clusters. The first and most significant cluster included observations of hydrogels from the perspective of self-assembly, swelling, and rheological properties, with the word hydrogel the most highlighted. The second cluster focused on encapsulation from the perspective of biopolymers, hydrogel particles, emulsions, and delivery systems. These two clusters showcase the transition from studies concerning the model properties of such hydrogels (cluster 2, before 2018) to the application of these hydrogels in tissue engineering, drug release and delivery, and the current application of self-assembly and self-healing hydrogels in food packaging (cluster 1, after 2019).

## 4. Hydrogel’s Induction Methods

**Figure 3:**
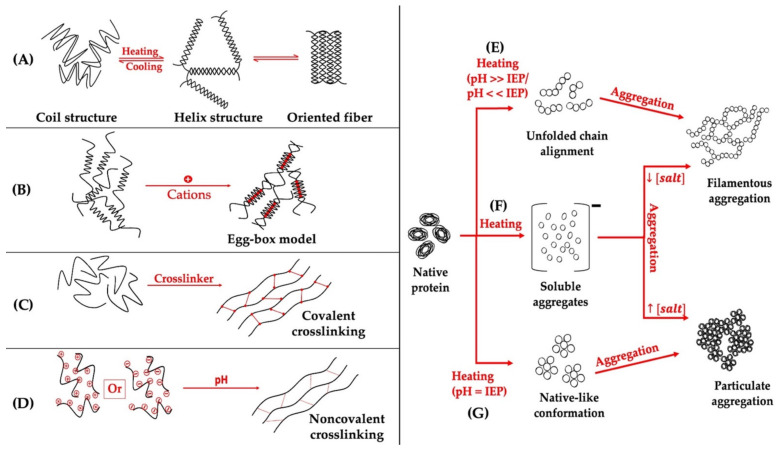
Illustration of the main mechanisms of formation of polysaccharides (**A**–**D**) and globular proteins hydrogel (**E**–**G**). (**A**) temperature-induced gelation of coil structure polysaccharides (e.g., κ-carrageenan), (**B**) ion-induced egg-box gelation of alginate, (**C**) covalent crosslinking-induced gelation (e.g., epichlorohydrin for cellulose hydrogel induction, glutaraldehyde for chitosan hydrogel induction), (**D**) pH-induced gelation (e.g., induction of pectin hydrogels), (**E**,**G**) temperature- and pH-induced globular protein gelation, (**F**) temperature- and ion-induced globular protein gelation [8,79,104].

Two factors need to be met to form a food hydrogel. The initial one is that the used biopolymer has hydrophilic groups, whereas the second one is the presence of crosslinking strength between the particles and molecules to initiate the aggregation process and the final formation of the network [1]. Figure 3 illustrates the main mechanisms of polysaccharides and proteins hydrogel formation. Based on the crosslinking mechanism of gelling, hydrogels can be divided into physically-, chemically-, enzymatically-, or multi-crosslinked. Physically crosslinked hydrogels are systems in which noncovalent interactions between the polymers are the precursor interactions that lead to the development of the structural network. Such physical mechanisms include electrostatic interactions [84], hydrogen bonds [85], crystallization [86], metal-ligand coordination [87], stereocomplex crystallization [88], hydrophobic interactions [89], conformation transformation [90], host-guest interaction [91], molecular specific binding [92], and π-π stacking [93]. Chemically crosslinked hydrogels are also known as “true gels”. They are obtained through the formation of covalent bonds between two polymers (Figure 3C). These kinds of junctions are usually non-reversible, permanent, and highly stable. Chemically crosslinked hydrogels can be obtained by free radical polymerization (pathway via monomers) [94] or by using crosslinkers, high-energy radiation, and the chemical reaction–pathway via polymers [95,96]. Enzymatically crosslinked hydrogels are obtained using enzymes such as trans-glutaminase [97], tyrosinase [98], laccase [99], horseradish peroxidase [100], etc. Notably, many hydrogels are obtained through multi-crosslinking mechanisms, using at least two described mechanisms depending on their structural complexity [101,102,103].

Through the years, many food hydrogel induction methods have been developed and applied in the food sectors [8,39,105]. The most conventional, well-studied methods of inducing proteins and polysaccharides gelation are pH, temperature, ion modulation (physical crosslinking methods), and enzymatic crosslinking. The recent development in the field of hydrogels brings new, unconventional induction methods, such as high-pressure and pulsed electric field [106,107]. The most crucial induction methods are discussed further below.

### 4.1. pH Induction

The pH induction is a cost-effective, simple, safe, and widely used food hydrogel induction method. By modulating the pH of the protein and/or polysaccharide dispersion, it is possible to affect the solubility, molecular conformation, and charge, as well as the zeta potential of the used biopolymers, altering the attractive and repulsive forces between particles, allowing the formation of intermolecular and intramolecular interactions that lead to the formation of the gel structure. Moreover, the conformational changes in the structure of proteins may occur [108,109]. Hydrogels obtained by pH induction can be utilized, among others, in the encapsulation of bioactive compounds. Zhan et al. [110] reported that it is possible to encapsulate curcumin in a zein-whey binary system using the pH-induced method. In other report, the pH-induced method was used to obtain an economical and environmentally friendly chitosan colloidal gel system with the potential for food or pharmaceutical formulations [111].

### 4.2. Heat Induction

This induction method is a “green” and environment-friendly method widely applied in food hydrogels. In the case of protein (globular proteins) hydrogels, the heat induction method involves two stages: protein unfolding (denaturation) or dissociate, and then the interaction and aggregation of the unfolded molecules caused by the interaction between their functional groups, allowing for the for the preparation of higher molecular weight complexes [112]. Lui et al. [113] reported in their study that they obtained a pectin-whey protein hydrogel with high structural strength and storage modulus by heat induction. Furthermore, Fu et al. [114] studied the heated-induced gelation of soy protein isolate at the subunit level. Depending on the polysaccharide structure and their source, a gel structure via heat induction can be produced, and examples may be cellulose (and its derivates) [115], curdlan [116], glucomannan [117], starch [118].

### 4.3. Ions Induction

The ions induction method, in some cases also known as cold induction (esp. in case of pre-denatured protein gel induction), is the addition of a salt ion (e.g., Na^+^, K^+^, Fe^3+^) to induce the formation of gel structure, which is also a very widely used method. The gelation process of the protein and polysaccharides can occur when the electrostatic repulsive interaction between the polymers is decreased or removed [119,120]. Recently, Zhou et al. [121] have reported that adding Na^+^ to a low-methoxyl pectin and soy protein dispersion affected the texture and viscoelastic properties of the cold-induced hydrogel. Additionally, they reported that only the addition of a low concentration of Na^+^ positively affected the studied properties. On the other hand, k-carrageenan gelation can be induced by adding K^+^ ions, as was studied by Chen et al. [122]. Additionally, it was demonstrated that it is possible to produce a composite hydrogel using chitosan and oxidized tannic acid by adding Fe^3+^ [123].

### 4.4. Enzymatic Induction

**Figure 4:**
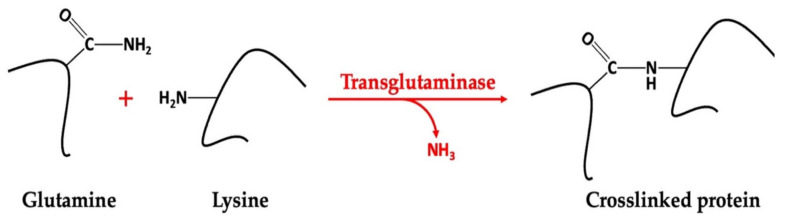
Schematic representation of protein crosslinking mechanism induced by transglutaminase [125,126].

By adding enzymes to biopolymers, it is possible to induce the formation of a hydrogel through a biochemical path in which the enzymes play the leading role in constructing the gel structure. Enzyme-induced gelation is based on the insertion of covalent crosslinks. The use of transglutaminase, which can induce protein gelation by promoting intramolecular and intermolecular crosslinking of the peptide chains (Figure 4), is one example of such an enzymatic induction [124].

The characteristic of transglutaminase-induced hydrogel crosslinking is related to the composition and conformation of the protein [127]. Transglutaminase has been effectively used in the induction of different types of proteins, such as soy protein [128], Bambara protein [129], as well as in the induction of binary-protein hydrogels composed of gelatin and carrageenan [130]. The other example can be protease, e.g., produced by Bacillus licheniformis, that can be used to induce the hydrolyzes α-Lactalbumin, which can then be used for the preparation of an amphiphilic peptide hydrogel used among others in the encapsulation of curcumin [131].

### 4.5. Freeze-Thaw Induction

**Figure 5:**
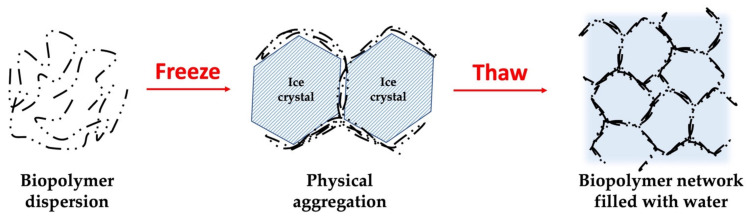
Schematic representation of freeze-thaw induction effect on cellulose nanocrystals hydrogel network formation [134,135].

This method involves freeze-thaw cycles, leading to phase separation and crystallization that affect the polysaccharide chain, allowing for the interaction between the chains by microcrystalline junction zones. This method is based on a repeated freezing process, storing in subzero temperatures, and thawing the dispersion in high temperatures [132]. Figure 5 represents the freeze-thaw induction method of cellulose nanocrystals. Xu et al. [133] studied β-glucan freeze-thaw gels as the carrier for the encapsulation of curcumin. They reported that these gels have great potential in developing natural drug delivery carriers. This induction method proved to be effective when it comes to thermolabile bioactive substances.

The freeze-thaw induction method proved very effective in regulating hydrogel’s textural properties while not negatively affecting its stability, even when two polymers were used in structure formation. This induction method was also demonstrated in research conducted by Shang et al. [136], where the effect of starch addition and freeze-thaw conditions on the water retention and texture properties of konjac glucomannan hydrogels was studied.

### 4.6. High Hydrostatic Pressure Induction

**Figure 6:**
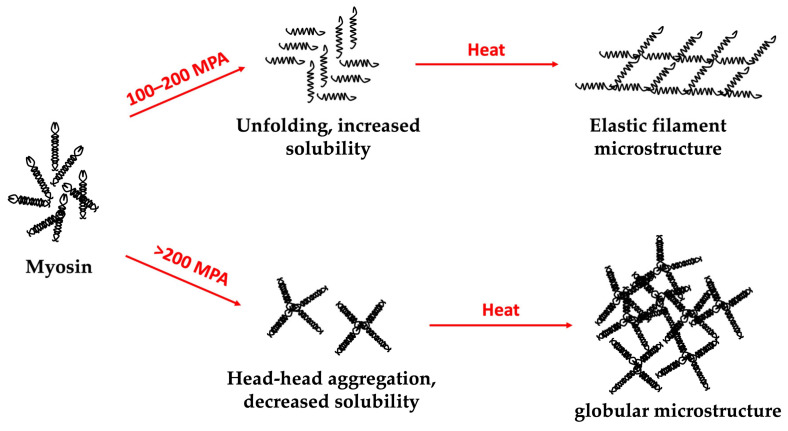
Schematic representation of HHP- and heat-induction effect on myosin hydrogel microstructure [139,140].

High hydrostatic pressure (HHP) induction is a novel method that has been extensively studied in terms of its ability to modify the physical properties of the protein and polysaccharide hydrogels. HHP provides the structural modification, aggregation, fragmentation that leads to gelatin production [137]. HHP can also transform protein structures by destroying the hydrophobic and electrostatic interactions, which influences denaturation, aggregation, and gelation. This induction technique can be used by itself or in combination with other induction methods (Figure 6), such as temperature induction [138].

Luo et al. [141] have studied the effect of HHP on the gelation behavior and microstructure of quinoa protein isolate dispersions. They found that using HHP induction allowed them to obtain hydrogels similar to the ones induced using heat treatment. Moreover, when using HHP, it is possible to obtain hydrogels at lower induction temperatures, which has excellent potential in incorporating thermolabile food compounds and nutraceuticals into the quinoa protein gel matrix. In a study conducted by Florowska et al. [28] regarding the effects of pressure level and time treatment of HHP on inulin gelation and properties of obtained hydrogels, the use of HHP pressure (higher than 300 MPa) was reported. The obtained hydrogels had higher stability and a more compressed and changed structure, which resulted in higher yield stress, lower spreadability, and more rigid and adhesive hydrogels. On the other hand, Liu et al. [142] stated that the induction of starch hydrogels using high pressure resulted in starch gels with different functional properties compared to those obtained by heat induction. The authors also reported that such a starch induction method might be of interest for food processing.

### 4.7. Pulsed Electric Field Induction

**Figure 7:**
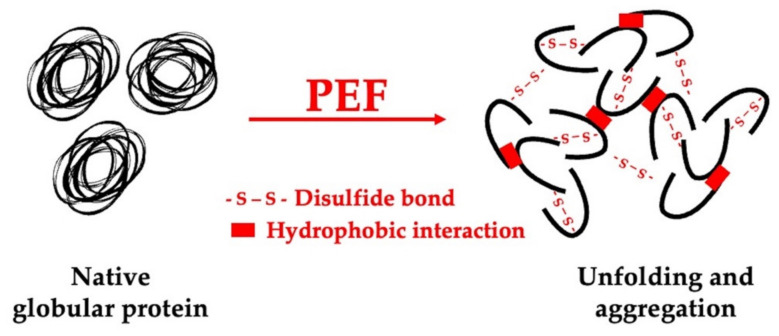
Schematic representation of PEF induction effect on globular protein [148,149].

Pulsed electric field (PEF) is a new physical method used to improve processes such as extraction, fermentation, dehydration, decontamination, etc. [143,144]. Figure 7 represents the effect of PEF on globular proteins. In addition, according to Giteru et al. [145], PEF treatment has the potential to be used to alter the functional properties of proteins and polysaccharides by inducing structural or conformational changes [146,147].

The use of a moderate pulsed electric field caused the structural unfolding of the myofibrillar protein of the porcine muscle, which resulted in the formation of a uniform and compact gel structure [150]. PEF treatment can also change myofibrillar protein hydrogels’ water distribution and mobility [151]. Moreover, the study conducted by Zhu et al. [152] on the use of the distributed electric field to induce the orientation of nanosheets resulted in the formation of complex anisotropic structures. These findings can be applied in the formation of hydrogels with biomimetic functionalities. PEF can be coupled with other induction techniques to design more complex hydrogels with specific functions [153].

## 5. Application Progress of Hydrogels as Food Matrices

Hydrogels present a wide range of properties (including high water content, flexibility, softness, and compatibility), making their application highly tunable for different food systems. Protein–polysaccharide composites have been so far successfully used only in the food packaging industry as they possess an oil barrier, water solubility, and tastelessness [154]. The commercially used edible films are produced mostly from cellulose and whey protein biopolymers [155], or alginate and collagen [156].

However, one of the critical characteristics of hydrogels is their similarity to living tissues, which can open new avenues for their use in food, particularly in the production of meat analogs [3]. Hydrogels can be used as base structures (matrices) when designing new food products since they can play a crucial role in achieving structure stability, sensory attributes, and nutritional aspects, such as being carriers for a wide range of nutrients and nutraceuticals [157].

Hydrogels have also been used successfully as fat mimetics in different food systems. Paglarini et al. [158] in their research demonstrated the potential of soy protein emulsion-filled hydrogel as a fat mimetic in frankfurter sausages. They reported that the sausages prepared using this emulsion-filled hydrogel exhibited the same hardness as traditional frankfurters. Moreover, Domínguez et al. [159] reported that the correctly chosen hydrogel formulation does not modify the sensory characteristics of meat products and allows for the reduction of both total fat and saturated fatty acids. Furthermore, the latest studies on hybrid gel prepared using canola oil/candelilla wax oleogel and gelatinized corn starch hydrogel also demonstrated the potential of hybrid hydrogels to be used as an alternative to commercial shortening to produce cookies with low-saturated fat content [160].

Recent research advances have recognized the utilization of bio-based biodegradable materials for food packaging to address the growing problem of the widespread use and misuse of petroleum-based polymeric materials [161]. Hydrogels prepared using biopolymers have great potential in manufacturing traditional, active, and intelligent food packaging. Hence, by embedding antimicrobial compounds (e.g., silver nanoparticles) into a hydrogel matrix, such a hydrogel can find use in the manufacturing of active packaging, which can reduce or inhibit the growth of harmful microorganisms [162]. Hydrogels can also be used to develop biosensors for intelligent food packaging, conveying information about a product’s freshness or the presence of contaminants [163,164,165].

The most recent trend in using hydrogels is the development of matrices that can replace animal-based food products in terms of texture and nutritional aspects. The food sector is increasingly becoming more concerned with providing enough nutritious food for everyone while protecting natural resources. That is why plant-based foods and hybrid food products (from animal and plant sources) are a new growing trend that can help with this sustainability challenge [65]. While developing new healthier foods using plant-based ingredients, the goal is to achieve the desired appearance, texture, flavor, mouthfeel, and functionality using healthy and sustainable plant-derived ingredients, such as lipids, proteins, and carbohydrates [65,166]. Additionally, plant-based products are often deficient in essential nutrients, such as vitamins (B_12_, D, etc.) and minerals (iron, zinc, etc.). As a result, there is a growing interest in fortifying such food systems with these nutrients. This fortification can be taken a step further by adding nutraceuticals such as carotenoids, curcuminoids, and polyphenols to improve the healthiness of these plant-based food systems. It is critical to comprehend how these ingredients can be integrated to form complex matrices resembling those found in animal-derived foods, as well as how the properties of these matrices affect the physicochemical and organoleptic properties of the final product [167]. Therefore, in this paper, the advancements in using hydrogels as bioactive substances carrying food matrices will be further discussed.

### 5.1. Encapsulation and Delivery Systems of Bioactive Compounds

Hydrogels are increasingly used as encapsulating and delivery agents because of their high encapsulation efficiency, biocompatibility, low cost, and environmentally friendly properties. These properties can be achieved due to their porous nature caused by the three-dimensional structures in which crosslinked polymers form large interstitial spaces that are densely packed with water. These interstitial spaces can also incorporate various nutrients and bioactive compounds [3]. That is why these spaces can be utilized to overcome some challenges related to adding health-beneficial substances to food products; for example, low thermal and chemical stability, poor solubility, and undesirable flavor organoleptic profile. Encapsulating the bioactive substances in hydrogels makes it possible to protect them from external environmental factors during production, storage, and even after consumption. Such factors include oxygen, heat, light, pH, enzymes, etc. [168,169,170].

**Figure 8:**
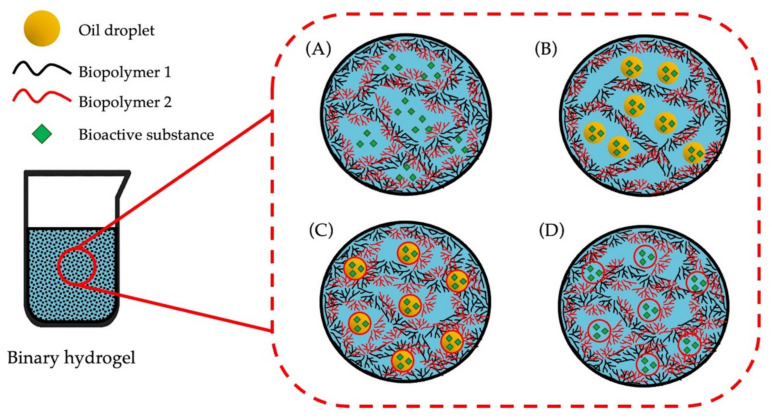
Schematic representation of the way in which bioactive substances can be embedded into a hydrogel matrix. (**A**) The hydrophilic bioactive substance is an inactive filler; (**B**) the hydrophobic bioactive substance is encapsulated in oil droplets and the oil droplets are inactive fillers. (**C**) the hydrophobic bioactive substance is encapsulated in oil droplets and the oil droplets are active fillers; (**D**) the hydrophilic bioactive substance is an active filler; Based on Farjami et al. [172], Liu et al. [41] and Li et al. [1].

Moreover, by mixing proteins and polysaccharides, it is possible to obtain improved structural and functional properties, which can be explained by the formation of protein–polysaccharide complexes via covalent and noncovalent interactions. These binary protein–polysaccharide hydrogels can be used as a matrix for embedding hydrophilic and hydrophobic compounds [171]. Hydrophobic compounds can be embedded into a hydrogel by first preparing an emulsion containing these bioactive substances and then introducing the biopolymers to the emulsion, resulting in an emulsion-filled hydrogel [172]. Both hydrophilic and hydrophobic compounds can either form the gel network, contributing to the strength and stability of the final hydrogel—such compounds are called active fillers (Figure 8C,D). However, the embedded compound might not interact or can interact minimally with the gel network—such compounds are called inactive fillers (Figure 8A,B).

Protein and polysaccharide hydrogels can be used as delivery systems for polyphenols, a group of compounds (over 8000 phenolic compounds) with a range of physiological functions, including antioxidant, anti-inflammatory, anti-virus, antibacterial, and immunity enhancement. These functional properties are mainly related to the phenolic groups and the conjugated double bonds [173]. Polyphenols are widely used in the food industry, but their bioavailability still imposes challenges because of their poor solubility and stability [174]. That is why many researchers are involved in designing a food-grade hydrogel carrier that can protect those compounds from oxygen, heat, light, and pH degradation. The latest finding regarding the use of hydrogels as delivery systems for phenolic compounds and vitamins are mentioned below.

Curcumin, a phenolic compound extracted from turmeric (*Curcuma longa* Linn.), has been well known for its health-promoting properties (antimicrobial, anti-inflammatory, antirheumatic, immunomodulatory, anti-carcinogenic). However, it exhibits poor water solubility and low bioavailability after ingestion [175]. Recently, proteins and polysaccharides-based hydrogels were developed to improve curcumin’s stability and bioavailability. George et al. [176], in their research on cellulose-chitosan-zinc oxide composite hydrogels for the encapsulation of curcumin, reported that the loading efficiency reached 89.68%. In addition, the obtained hydrogel exhibited an antimicrobial effect on Trichophyton rubrum and Staphylococcus aureus and a controlled release at pH 7.4. In another study, curcumin was embedded in a chitosan/lotus root pectin hydrogel with an efficiency of 90.3% and improved solubility and stability [173]. Moreover, a nanoparticles-in-microparticles hydrogel system was fabricated by electrospray technology for curcumin colon-targeting oral delivery, which enabled curcumin release and entry to the macrophages [177]. Kour et al. [178] studied the effect of nanoemulsion-loaded hybrid biopolymeric hydrogel beads on the release kinetics, antioxidant potential, and antibacterial activity of encapsulated curcumin. They found that the high structural stability of the obtained carriers and their effective delivery of curcumin can provide a novel and tailored formulation out of polymers for oral drug delivery.

Epigallocatechin gallate (EGGG) is a catechin phenolic active compound with several health-beneficial properties, such as antioxidant, anti-tumor, antiviral, antibacterial, and cardio cerebral vessel protective. The polyhydroxy structure of catechins makes them unstable in neutral and alkaline pH. Additionally, they can be glucosylated or methylated by gastrointestinal tract enzymes, making them highly unstable and biologically unavailable [179]. To improve the stability and release of EGGG, Wang et al. [180] prepared a composite protein–polysaccharide hydrogel using carboxymethyl konjac glucomannan and gelatin. Authors reported that obtained hydrogels had better pH-sensitive properties, which enhanced the encapsulation and the bioavailability of EGGG. Furthermore, Yu et al. [181] reported that EGGG added to collagen hydrogels acted as an active filler by narrowing the pore size and strengthening the collagen fiber network. This effect was due to the formation of covalent bonds between lysine and EGCG. What is more, the incorporation of nanofiber particles coated with epigallocatechin-gallate (EGCG) into gelatin methacryloyl hydrogel reduced the free-radical-derived cellular damage when using 3D tissue fabrication (ex vivo) [182]. Wu et al. [183] demonstrated that using konjac galactomannan with the addition of oxidized hyaluronic acid enhances the stability and control release of EGGG. Other studies also reported the positive effect of EGGG on the structural remodeling of soy protein-derived amyloid fibrils hydrogel [184].

Resveratrol is another poorly water-soluble polyphenolic compound that exhibits various physiological properties (e.g., oxidative stress, anti-inflammatory, anti-obesity, anti-cancer, etc.) [185]. Additionally, to its poor water solubility, resveratrol is characterized by a fast metabolism in the gastrointestinal environment, which affects bioavailability. Fan et al. [186] prepared pea protein particles with calcium-induced cross-linking in which they encapsulated resveratrol. This encapsulation led to enhancing the physicochemical stability of the compounds, as well as led to a better antioxidant ability. Other studies on the improvement of resveratrol stability included the preparation of a resveratrol-loaded nanostructured lipid carrier hydrogel that significantly enhanced anti-UV irradiation and anti-oxidative activity in vitro and in vivo [187]. Currently, Pickering emulsion presents a high potential in the encapsulation of resveratrol. Based on Wu et al.’s [188] reports, it is possible to conclude that Pickering emulsion prepared using sodium alginate and pectin has a promising potential in developing low-calorie food products while contributing to the delivery of resveratrol to the gastrointestinal tract.

Anthocyanins are water-soluble flavonoids with high antioxidant activity. Their use in the food industry is limited due to their rapid degradation triggered by the pH value. They also have a low bioavailability and recovery rate after ingestion because of their low resistance to environmental changes [189]. Additionally, Jin et al. [190], in their study, prepared a konjac glucomannan and xanthan gum hydrogel in which they embedded anthocyanins. They reported that this synergistic hydrogel enhanced the thermal stability of anthocyanins at various pH values (3.0, 6.0, and 9.0). Ćorković et al. [191] also reported that the use of carboxymethylcellulose hydrogel as polyphenol carriers, specifically anthocyanins, helped preserve their antioxidant capacity. These findings showcased that proper formulation of food hydrogel, including the proper selection of biopolymers, can significantly maximize the retention of anthocyanins. In the current study conducted by Liu et al. [192], it was reported that the efficiency of anthocyanin encapsulation in gelatin/gellan hydrogel was high because of the high density of the formed structure. Moreover, the gelatin/gellan hydrogel protected the embedded anthocyanins during digestion, increasing its bioavailability in the small intestine. However, the proper selection of hydrogel building components is critical because anthocyanins may be degraded rather than protected, as observed in the studies of Kopjar et al. [193], in which the fortification of anthocyanins-loaded pectin hydrogel with apple fibers caused a substantial degradation in the retention of the anthocyanins. Furthermore, hydrogel loaded with anthocyanins can also be utilized as a colorimetric pH indicator to monitor, for example, the freshness of food products [166,194,195].

Quercetin, a flavonoid with beneficial properties, such as exhibited antioxidant, anti-inflammatory, anticancer, and cardioprotective, also exhibits low solubility and physicochemical instability, making it hard to be absorbed and utilized by the human body [196]. Several hydrogel systems have been recently prepared to protect this compound from the environment and raise its bioavailability. Quercetin-loaded pH-sensitive gellan gum hydrogels were induced using an ionotropic gelation method, and it was found that the obtained hydrogel beads had a pH-responsive release behavior. This release behavior improved the intestinal stability of this bioactive substance [35]. Moreover, Liu et al. [197] developed a lotus root amylopectin-coated whey protein hydrogel to protect quercetin. They reported that the obtained hydrogel enhanced the stability of quercetin while improving its bioavailability (in mice). In another study, linseed oil and quercetin were co-loaded to liposome-chitosan hydrogel beads. Based on the obtained results, the authors found that the chemical stability of quercetin could be improved by loading liposomes into hydrogel beads [198]. Moreover, Hu et al. [199] studied the co-encapsulation of epigallocatechin and quercetin in double-emulsion hydrogel beads and reported that obtained hydrogel beads inhibited oil digestion while increasing quercetin bioavailability.

Hydrogels obtained using food-grade biopolymers (proteins and polysaccharides) have been utilized for vitamin protection and delivery. The complexation of vitamin A and milk protein has been proven to increase the water-solubility and the light and heat stability of this vitamin [200]. Moreover, Rana et al. [201] also reported that vitamin A-loaded caseinate complexes improved vitamin A bioavailability. Similarly, Kaur et al. [202] highlighted the potential of chitosan and gelatin-based hydrogel to deliver vitamin B_1_. A chemically crosslinked cellulose–hemicellulose-based vitamin B_12_-loaded hydrogel was also reported to be effective in releasing this vitamin when the in vitro release is performed in successive buffers (from pH 1.2 to 7.4) [203]. Furthermore, β-cyclodextrin-soy soluble polysaccharide-based hydrogel was used to encapsulate and deliver vitamin E, showcasing the tunability of the swelling release properties of this vitamin both in-vitro and in-vivo [204]. Moreover, Martinez et al. [205] reported that the incorporation of vitamin E into a bigel (a combination of a hydrogel and an organogel) increased the diameter of the inner phase and the strength of the obtained structure. Mir et al. [206], in their research on glycerol-crosslinked guar gum monoaldehyde-based superabsorbent hydrogels for vitamin B_6_, concluded that the release of vitamin B_6_ depended on the pH of the medium (at pH 7, the concentration of the released vitamin was 79.2%).

### 5.2. Bioactive Substances Targeted Transport and Controlled Release

Because of the ability of hydrogels to hold large amounts of water or biological fluids, they can be used as carriers for bioactive substances, which can be embedded in the 3D hydrogel’s structure. Hydrogels have significant potential in developing targeted release systems, which can release the embedded substances into the digestive tract. When choosing biopolymers such as building blocks, what needs to be taken into consideration is their digestibility [207,208,209]. Proteins are known to be very efficiently digestible because of multiple peptidases in the digestive system. Additionally, denatured proteins in hydrogels obtained using heat induction are even more digestible [210]. On the other hand, polysaccharides have diverse digestion pathways, which depend on their type. For example, starch digestibility varies from rapidly digestible to indigestible. Some starches can be rapidly hydrolyzed by amylase in the mouth or the small intestine [211]. However, some polysaccharides, such as inulin, pectin, alginate, etc., can only be fermented by the microbiota in the colon [212,213].

**Figure 9:**
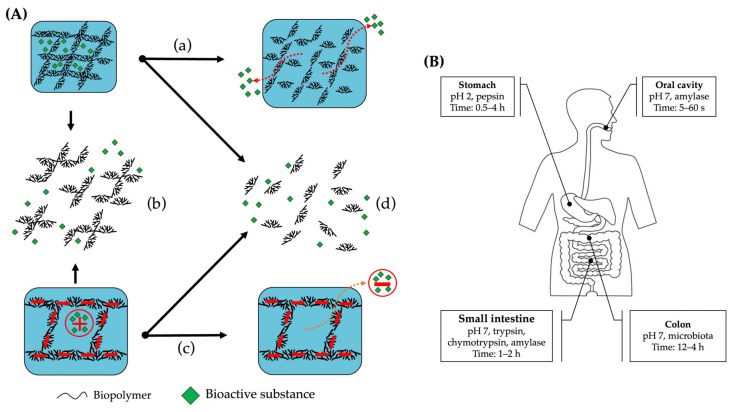
Food hydrogel and the digestive system interaction. (**A**) Potential pathways for targeted compound release from hydrogels: (**a**) swelling; (**b**) disintegration; (**c**) molecular interaction; (**d**) rrosion. (**B**) Schematic representations of physiological conditions (pH, enzyme, and retention time) of the gastrointestinal tract [207,216,217].

Binary protein–polysaccharide hydrogels that deliver bioactive compounds to specific areas of the digestive tract can be developed based on the properties of the biopolymers used as hydrogel building blocks. These hydrogels can be designed to deliver the bioactive substance in the right place and time under the influence of factors such as pH, temperature, enzyme, or microbiota. These factors affect the hydrogel’s 3D structure, leading to its swelling or shrinkage and the release of the compound [214,215]. Based on the physiological conditions in different parts of the human digestive tract, it is possible to design a suitable hydrogel to deliver the bioactive compound to the targeted delivery site. The embedded bioactive substances can be released (Figure 9A) via swelling (change in volume), disintegration (dissociation of electrostatic coacervates), change in the molecular interactions (e.g., change in the electrostatic interaction between the bioactive compound and the polymeric building blocks), erosion (fermentation by the microbiota, digestion by enzymes) of the hydrogel’s carriers [216]. For the hydrogels to deliver the embedded compound to the oral cavity, stomach, or small intestine, they should be pH- and enzyme-sensitive (Figure 9B). When the targeted site is the colon, the used hydrogel should be pH-sensitive and fermentable by the microbiota [208].

Certain hydrogels can respond to chemical changes in the pH and ionic composition in the environment surrounding them. This response leads to changes in the structure of the polymer network. Such hydrogels are called pH- and ion-responsive [218]. Xie et al. [219] reported that they synthesized a hydrogel using Chinese quince seed gum, which has promising potential for the oral delivery of drugs. Furthermore, Sarıyer et al. [220] developed pH-responsive alginate and κ-carrageenan hydrogels for the targeted release of bovine serum albumin. The targeted delivery of albumin to the intestines was achieved through diffusion and polymer structure relaxation. Temperature-responsive hydrogels are another type of carrier that respond to the changes in the temperature of the environment they are in by swelling or shrinking, which allows for the bioactive compounds to be released from the gel structure [221]. Temperature-responsive hydrogels might not be used to deliver bioactive substances to the stomach, small intestine, and colon but instead for oral (buccal) delivery. The such hydrogel can be developed to release the embedded substance at a temperature of 37 °C. Baus et al. [222] assessed in-vitro methods for the characterization of mucoadhesive hydrogels prepared using biopolymers, such as hydroxyethyl cellulose, carboxymethyl cellulose, xanthan gum, hyaluronic acid, and sodium alginate. They found out that xanthan gum had the highest resistance to the removal by artificial saliva. They also reported that based on the residence time of hydrogels, it is possible to develop a formulation with the best mucoadhesive properties for the delivery of bioactive compounds to the buccal area. Another type of hydrogel undergoes changes in its structure because of the activity of a specific enzyme. These hydrogels are enzyme-responsive and can be used to deliver a compound to a specific region of the digestive tract—where the concentration of enzymes, such as proteases or amylases, are the highest. The microbiota can also release the embedded compounds since it also produces enzymes that are not produced by the human gastrointestinal tract and can hydrolyze specific bonds of the biopolymers present in the 3D structure of the hydrogel. Wang et al. [223] developed an intestine enzyme-responsive polysaccharide-based hydrogel using carboxymethyl chitosan embedded with an antitumor-selective kinase inhibitor. They reported that the obtained hydrogel was able to enhance the therapeutic efficiency.

Because of the wide range of possibilities in developing protein–polysaccharide hydrogels, it is possible to design hydrogels that can be responsive to multiple stimuli depending on the targeted delivery area. Zhao and Li [224] obtained pH- and temperature-responsive hydrogels using Tremella polysaccharides, carboxymethyl cellulose, and nonionic surfactants as the main hydrogel building blocks. Whereas Liao and Huang [225] obtained a pH- and magnetic-responsive hydrogel using carboxymethyl chitin, for which the swelling structure degree can be regulated depending on the concentration levels of Fe_3_O_4_, the release mechanism is triggered by pH modulation.

## 6. Concluding Remarks and Future Perspectives

Protein–polysaccharide hydrogels have great potential for overcoming the limitations of hydrogels prepared with a single biopolymer, such as poor water-holding capacity and gel strength, as well as physical instability. In this review, we conducted a bibliometric analysis to characterize research trends in food protein–polysaccharide hydrogels (over the last ten years). We also discussed the latest development in conventional methods of inducing proteins and polysaccharides gelation (pH, temperature, ions modulation, and enzymatic crosslinking) and the new, unconventional induction methods, such as high-pressure and pulsed electric field treatment. Additionally, the newest developments regarding the application of hydrogels as food matrices, specifically as carriers for the targeted delivery of bioactive compounds, were discussed.

The studies regarding protein–polysaccharide hydrogels in food science are still minimal. This knowledge gap allows for new findings to be implemented in developing novel hydrogels for food applications. This hydrogel development can be achieved through a cross-integrated multidisciplinary approach between the food industry and other industry areas with advanced hydrogel knowledge (pharmaceutical, biomedical).

Protein–polysaccharide hydrogels have a promising potential in food applications by improving the stability and increasing the nutritious value of food systems while building a structural matrix that can be utilized as non-invasive bioactive compounds- targeted delivery systems. These highly tunable hydrogel properties can allow for the development of new, health-promoting plant-based or hybrid food systems that provide consumers with all the necessary nutrients based on their physiological needs. Therefore, there is considerable room for further research in a wide range of food hydrogel applications. There is a particular need to assess the possibility of using building blocks, such as plant-based proteins and polysaccharides, to develop a food hydrogel matrix that will protect the bioactive compound during processing, storage, and digestion, while increasing the bioavailability of these bioactive substances in the specific targeted area of the digestive system.

## Figures and Tables

**Table 1:** The 10 most cited original research papers on the topic of binary hydrogels from 2012 to 2022.

**Sr. No.**	**Material**	**Induction Method**	**Key Findings**	**Applications**	**Ref.**
1	Xanthan gum/β-lactoglobulin	pH (4.4)	The complexation between the polymers resulted in a functional hydrogel, in which the structure strength mainly depended on xanthan gum.	Encapsulation of bioactive molecules	[70]
2	Whey protein/pectin Whey protein/alginate Whey protein/xanthan	Heat (90 °C)	The study provided information on the release mechanism of the obtained emulsion-filled hydrogels. Whey protein/pectin hydrogel had the highest release exponent.	Bioactive compounds delivery matrices	[71]
3	Gelatin/glucan	Heat (45–120 °C)	In comparison with pure gelatin the gelatin/glucan hydrogel exhibited improved mechanical properties.	Food and pharmaceutical	[72]
4	Konjac glucomannan/gum tragacanth	Heat (60 °C)	The obtained hydrogel was formed mainly by hydrogen bonding. The hydrogel exhibited a significant thermosensitive behavior between 35–45 °C.	Thermosensitive delivery system	[73]
5	Gelatin/tara gum	pH (3.5–11.0) and ion (salt: 0–300 mmol/L)	A synergistic effect of tara gum of the gelatin gel structure was observed. The hydrogel formation was not affected by the pH. The addition of salt (50 mmol/L) had the most significant on the mechanical attributes.	Food rheology modulation and delivery system	[74]
6	Caseinate/pectin	Enzymatic (transglutaminase)	The hydrogels obtained using enzymatic crosslinking exhibited significant integrity under pH ranging from 6 to 8. Both enzymatically crosslinked and not crosslinked network displayed a high stability to heating and low pH.	Delivery matrices for lipophilic bioactives	[75]
7	Gellan gum/collagen	Heat (90 °C)	A new process of gelation was proposed, which is based on dripping the gellan-anthocyanin dispersion into the cold (10 °C) collagen dispersion. The obtained network exhibited high anthocyanin retention (>84%).	Encapsulation of bioactive molecules	[76]
8	Starch/alginate	Ion (CaCl_2_)	The retention of insulin was >80%. The obtained hydrogel exhibited promising properties in terms of safe delivery of insulin via oral pathway.	Insulin oral delivery system	[77]
9	Soy protein/κ-carrageenan	Heat (80 °C)	The hydrogel with the addition of 0.6% κ-carrageenan displayed the most dense and uniform structure. Additionally, κ-carrageenan protected the soy protein and the embedded flax lignans from erosion caused by digestive enzymes.	Carriers for water-soluble bioactive compounds	[78]
10	Alginate/inulin Chitosan/inulin	Heat (80–90 °C)	The addition of alginate or chitosan had no significant impact on the gelling ability of inulin. Chitosan (0.5 g/100 g) addition improved the stability of the obtained hydrogels.	Functional ingredient for developing new health-promoting food products	[29]

## References

[1] Li J., Jia X., Yin L. (2021). Hydrogel: Diversity of Structures and Applications in Food Science. Food Rev. Int..

[2] Khalesi H., Lu W., Nishinari K., Fang Y. (2020). New Insights into Food Hydrogels with Reinforced Mechanical Properties: A Review on Innovative Strategies. Adv. Colloid Interface Sci..

[3] Gul K., Gan R.-Y., Sun C.-X., Jiao G., Wu D.-T., Li H.-B., Kenaan A., Corke H., Fang Y.-P. (2021). Recent Advances in the Structure, Synthesis, and Applications of Natural Polymeric Hydrogels. Crit. Rev. Food Sci. Nutr..

[4] Singh V., Prasad Y.S., Rachamalla A.K., Rebaka V.P., Banoo T., Maheswari C.U., Sridharan V., Lalitha K., Nagarajan S. (2022). Hybrid Hydrogels Derived from Renewable Resources as a Smart Stimuli Responsive Soft Material for Drug Delivery Applications. RSC Adv..

[5] Light K., Karboune S. (2021). Emulsion, Hydrogel and Emulgel Systems and Novel Applications in Cannabinoid Delivery: A Review. Crit. Rev. Food Sci. Nutr..

[6] Zhang M., Yan S., Xu X., Yu T., Guo Z., Ma M., Zhang Y., Gu Z., Feng Y., Du C. (2021). Three-Dimensional Cell-Culture Platform Based on Hydrogel with Tunable Microenvironmental Properties to Improve Insulin-Secreting Function of MIN6 Cells. Biomaterials.

[7] Kaith B.S., Singh A., Sharma A.K., Sud D. (2021). Hydrogels: Synthesis, Classification, Properties and Potential Applications—A Brief Review. J. Polym. Environ..

[8] Cao Y., Mezzenga R. (2020). Design Principles of Food Gels. Nat. Food.

[9] Klein M., Poverenov E. (2020). Natural Biopolymer-based Hydrogels for Use in Food and Agriculture. J. Sci. Food Agric..

[10] Zhu P., Huang W., Guo X., Chen L. (2021). Strong and Elastic Pea Protein Hydrogels Formed through PH-Shifting Method. Food Hydrocoll..

[11] Liu J., Li Z., Lin Q., Jiang X., Yao J., Yang Y., Shao Z., Chen X. (2018). A Robust, Resilient, and Multi-Functional Soy Protein-Based Hydrogel. ACS Sustain. Chem. Eng..

[12] Ingrassia R., Palazolo G.G., Wagner J.R., Risso P.H. (2019). Heat Treatments of Defatted Soy Flour: Impact on Protein Structure, Aggregation, and Cold-Set Gelation Properties. Food Struct..

[13] Moreno H.M., Domínguez-Timón F., Díaz M.T., Pedrosa M.M., Borderías A.J., Tovar C.A. (2020). Evaluation of Gels Made with Different Commercial Pea Protein Isolate: Rheological, Structural and Functional Properties. Food Hydrocoll..

[14] Wang B., Liu F., Luo S., Li P., Mu D., Zhao Y., Zhong X., Jiang S., Zheng Z. (2019). Effects of High Hydrostatic Pressure on the Properties of Heat-Induced Wheat Gluten Gels. Food Bioprocess Technol..

[15] Zhang Z., Chen X., Liu X., Liu W., Liu Q., Huang J., Zhang L., Hu H. (2022). Effect of Salt Ions on Mixed Gels of Wheat Gluten Protein and Potato Isolate Protein. LWT.

[16] Gagliardi A., Voci S., Paolino D., Fresta M., Cosco D. (2020). Influence of Various Model Compounds on the Rheological Properties of Zein-Based Gels. Molecules.

[17] Gagliardi A., Froiio F., Salvatici M.C., Paolino D., Fresta M., Cosco D. (2020). Characterization and Refinement of Zein-Based Gels. Food Hydrocoll..

[18] Wagner J., Biliaderis C.G., Moschakis T. (2020). Whey Proteins: Musings on Denaturation, Aggregate Formation and Gelation. Crit. Rev. Food Sci. Nutr..

[19] Gao X., Yao Y., Wu N., Xu M., Zhao Y., Tu Y. (2020). The Sol-Gel-Sol Transformation Behavior of Egg White Proteins Induced by Alkali. Int. J. Biol. Macromol..

[20] Manzoor A., Dar A.H., Pandey V.K., Shams R., Khan S., Panesar P.S., Kennedy J.F., Fayaz U., Khan S.A. (2022). Recent Insights into Polysaccharide-Based Hydrogels and Their Potential Applications in Food Sector: A Review. Int. J. Biol. Macromol..

[21] Berton S.B.R., de Jesus G.A.M., Sabino R.M., Monteiro J.P., Venter S.A.S., Bruschi M.L., Popat K.C., Matsushita M., Martins A.F., Bonafé E.G. (2020). Properties of a Commercial κ-Carrageenan Food Ingredient and Its Durable Superabsorbent Hydrogels. Carbohydr. Res..

[22] Akalin G.O., Pulat M. (2020). Preparation and Characterization of κ-Carrageenan Hydrogel for Controlled Release of Copper and Manganese Micronutrients. Polym. Bull..

[23] Malik N.S., Ahmad M., Minhas M.U., Tulain R., Barkat K., Khalid I., Khalid Q. (2020). Chitosan/Xanthan Gum Based Hydrogels as Potential Carrier for an Antiviral Drug: Fabrication, Characterization, and Safety Evaluation. Front. Chem..

[24] Ma L., Tan Y., Chen X., Ran Y., Tong Q., Tang L., Su W., Wang X., Li X. (2022). Injectable Oxidized Alginate/Carboxylmethyl Chitosan Hydrogels Functionalized with Nanoparticles for Wound Repair. Carbohydr. Polym..

[25] Hu C., Lu W., Mata A., Nishinari K., Fang Y. (2021). Ions-Induced Gelation of Alginate: Mechanisms and Applications. Int. J. Biol. Macromol..

[26] Florowska A., Hilal A., Florowski T., Mrozek P., Wroniak M. (2022). Sodium Alginate and Chitosan as Components Modifying the Properties of Inulin Hydrogels. Gels.

[27] Michalska-Ciechanowska A., Majerska J., Brzezowska J., Wojdyło A., Figiel A. (2020). The Influence of Maltodextrin and Inulin on the Physico-Chemical Properties of Cranberry Juice Powders. ChemEngineering.

[28] Florowska A., Florowski T., Sokołowska B., Adamczak L., Szymańska I. (2021). Effects of Pressure Level and Time Treatment of High Hydrostatic Pressure (HHP) on Inulin Gelation and Properties of Obtained Hydrogels. Foods.

[29] Larrea-Wachtendorff D., Sousa I., Ferrari G. (2021). Starch-Based Hydrogels Produced by High-Pressure Processing (HPP): Effect of the Starch Source and Processing Time. Food Eng. Rev..

[30] Zeng S., Zhang J., Zu G., Huang J. (2021). Transparent, Flexible, and Multifunctional Starch-Based Double-Network Hydrogels as High-Performance Wearable Electronics. Carbohydr. Polym..

[31] Wang L., Hu S., Ullah M.W., Li X., Shi Z., Yang G. (2020). Enhanced Cell Proliferation by Electrical Stimulation Based on Electroactive Regenerated Bacterial Cellulose Hydrogels. Carbohydr. Polym..

[32] Hu Y., Zhang M., Qin C., Qian X., Zhang L., Zhou J., Lu A. (2021). Transparent, Conductive Cellulose Hydrogel for Flexible Sensor and Triboelectric Nanogenerator at Subzero Temperature. Carbohydr. Polym..

[33] Mittal H., Morajkar P.P., al Alili A., Alhassan S.M. (2020). In-Situ Synthesis of ZnO Nanoparticles Using Gum Arabic Based Hydrogels as a Self-Template for Effective Malachite Green Dye Adsorption. J. Polym. Environ..

[34] Makar L.E., Nady N., Abd El-Fattah A., Shawky N., Kandil S.H. (2022). Unmodified Gum Arabic/Chitosan/Nanohydroxyapatite Nanocomposite Hydrogels as Potential Scaffolds for Bone Regeneration. Polymers.

[35] Dey M., Ghosh B., Giri T.K. (2020). Enhanced Intestinal Stability and PH Sensitive Release of Quercetin in GIT through Gellan Gum Hydrogels. Colloids Surf. B Biointerfaces.

[36] Das M., Giri T.K. (2020). Hydrogels Based on Gellan Gum in Cell Delivery and Drug Delivery. J. Drug Deliv. Sci. Technol..

[37] Zha F., Rao J., Chen B. (2021). Plant-Based Food Hydrogels: Constitutive Characteristics, Formation, and Modulation. Curr. Opin. Colloid Interface Sci..

[38] Fan Z., Cheng P., Zhang P., Zhang G., Han J. (2022). Rheological Insight of Polysaccharide/Protein Based Hydrogels in Recent Food and Biomedical Fields: A Review. Int. J. Biol. Macromol..

[39] Akhtar M., Ding R. (2017). Covalently Cross-Linked Proteins & Polysaccharides: Formation, Characterisation and Potential Applications. Curr. Opin. Colloid Interface Sci..

[40] Huang Q., Liu Z., Pei Y., Li J., Li B. (2021). Gelation Behaviors of the Konjac Gum from Different Origins: *A. guripingensis* and *A. rivirei*. Food Hydrocoll..

[41] Liu K., Chen Y.Y., Zha X.Q., Li Q.M., Pan L.H., Luo J.P. (2021). Research Progress on Polysaccharide/Protein Hydrogels: Preparation Method, Functional Property and Application as Delivery Systems for Bioactive Ingredients. Food Res. Int..

[42] Semenova M. (2017). Protein–Polysaccharide Associative Interactions in the Design of Tailor-Made Colloidal Particles. Curr. Opin. Colloid Interface Sci..

[43] Lavoisier A., Aguilera J.M. (2019). Starch Gelatinization inside a Whey Protein Gel Formed by Cold Gelation. J. Food Eng..

[44] Tang M.X., Zhu Y.D., Li D., Adhikari B., Wang L.J. (2019). Rheological, Thermal and Microstructural Properties of Casein/κ-Carrageenan Mixed Systems. LWT.

[45] Zernov A., Baruch L., Machluf M. (2022). Chitosan-Collagen Hydrogel Microparticles as Edible Cell Microcarriers for Cultured Meat. Food Hydrocoll..

[46] Ingrassia R., Bea L.L., Hidalgo M.E., Risso P.H. (2019). Microstructural and Textural Characteristics of Soy Protein Isolate and Tara Gum Cold-Set Gels. LWT.

[47] Yang X., Li A., Li D., Guo Y., Sun L. (2021). Applications of Mixed Polysaccharide-Protein Systems in Fabricating Multi-Structures of Binary Food Gels—A Review. Trends Food Sci. Technol..

[48] Shahbazizadeh S., Naji-Tabasi S., Shahidi-Noghabi M. (2022). Development of Soy Protein/Sodium Alginate Nanogel-Based Cress Seed Gum Hydrogel for Oral Delivery of Curcumin. Chem. Biol. Technol. Agric..

[49] Zhang Q., Gu L., Su Y., Chang C., Yang Y., Li J. (2021). Development of Soy Protein Isolate/κ-Carrageenan Composite Hydrogels as a Delivery System for Hydrophilic Compounds: Monascus Yellow. Int. J. Biol. Macromol..

[50] Florowska A., Hilal A., Florowski T., Wroniak M. (2020). Addition of Selected Plant-Derived Proteins as Modifiers of Inulin Hydrogels Properties. Foods.

[51] Yan W., Zhang B., Yadav M.P., Feng L., Yan J., Jia X., Yin L. (2020). Corn Fiber Gum-Soybean Protein Isolate Double Network Hydrogel as Oral Delivery Vehicles for Thermosensitive Bioactive Compounds. Food Hydrocoll..

[52] Wang Y., Jiao A., Qiu C., Liu Q., Yang Y., Bian S., Zeng F., Jin Z. (2022). A Combined Enzymatic and Ionic Cross-Linking Strategy for Pea Protein/Sodium Alginate Double-Network Hydrogel with Excellent Mechanical Properties and Freeze-Thaw Stability. Food Hydrocoll..

[53] Zhan F., Shi M., Wang Y., Li B., Chen Y. (2019). Effect of Freeze-Drying on Interaction and Functional Properties of Pea Protein Isolate/Soy Soluble Polysaccharides Complexes. J. Mol. Liq..

[54] Kaushik P., Priyadarshini E., Rawat K., Rajamani P., Bohidar H.B. (2020). PH Responsive Doxorubucin Loaded Zein Nanoparticle Crosslinked Pectin Hydrogel as Effective Site-Specific Anticancer Substrates. Int. J. Biol. Macromol..

[55] Siddiqui S.A., Alvi T., Biswas A., Shityakov S., Gusinskaia T., Lavrentev F., Dutta K., Khan M.K.I., Stephen J., Radhakrishnan M. (2022). Food Gels: Principles, Interaction Mechanisms and Its Microstructure. Crit. Rev. Food Sci. Nutr..

[56] Rodríguez-Rojas A., Arango Ospina A., Rodríguez-Vélez P., Arana-Florez R. (2019). ¿What Is the New about Food Packaging Material? A Bibliometric Review during 1996–2016. Trends Food Sci. Technol..

[57] Moreira M.N.B., da Veiga C.P., da Veiga C.R.P., Reis G.G., Pascuci L.M. (2022). Reducing Meat Consumption: Insights from a Bibliometric Analysis and Future Scopes. Future Foods.

[58] Hallinger P., Kovačević J. (2019). A Bibliometric Review of Research on Educational Administration: Science Mapping the Literature, 1960 to 2018. Rev. Educ. Res..

[59] Araújo A.G., Pereira Carneiro A.M., Palha R.P. (2020). Sustainable Construction Management: A Systematic Review of the Literature with Meta-Analysis. J. Clean. Prod..

[60] Batistič S., Černe M., Vogel B. (2017). Just How Multi-Level Is Leadership Research? A Document Co-Citation Analysis 1980–2013 on Leadership Constructs and Outcomes. Leadersh. Q..

[61] Park J.Y., Nagy Z. (2018). Data on the Interaction between Thermal Comfort and Building Control Research. Data Brief.

[62] Sweileh W.M., Al-Jabi S.W., Zyoud S.H., Sawalha A.F., Abu-Taha A.S. (2018). Global Research Output in Antimicrobial Resistance among Uropathogens: A Bibliometric Analysis (2002–2016). J. Glob. Antimicrob. Resist..

[63] Han L., Gong Z. (2021). Visual Analysis of Construction Waste Research Based on VOSviewer. E3S Web Conf..

[64] Yu Y., Li Y., Zhang Z., Gu Z., Zhong H., Zha Q., Yang L., Zhu C., Chen E. (2020). A Bibliometric Analysis Using VOSviewer of Publications on COVID-19. Ann. Transl. Med..

[65] Peschel A.O., Kazemi S., Liebichová M., Sarraf S.C.M., Aschemann-Witzel J. (2019). Consumers’ Associative Networks of Plant-Based Food Product Communications. Food Qual. Prefer..

[66] Ali A., Ahmed S. (2018). Development of Hydrogels from Edible Polymers. Polymers for Food Applications.

[67] Wang M., Li D., Zang Z., Sun X., Tan H., Si X., Tian J., Teng W., Wang J., Liang Q. (2021). 3D Food Printing: Applications of Plant-Based Materials in Extrusion-Based Food Printing. Crit. Rev. Food Sci. Nutr..

[68] Wollschlaeger J.O., Maatz R., Albrecht F.B., Klatt A., Heine S., Blaeser A., Kluger P.J. (2022). Scaffolds for Cultured Meat on the Basis of Polysaccharide Hydrogels Enriched with Plant-Based Proteins. Gels.

[69] Chen C., Chitose A., Kusadokoro M., Nie H., Xu W., Yang F., Yang S. (2021). Sustainability and Challenges in Biodiesel Production from Waste Cooking Oil: An Advanced Bibliometric Analysis. Energy Rep..

[70] Le X.T., Turgeon S.L. (2013). Rheological and Structural Study of Electrostatic Cross-Linked Xanthan Gum Hydrogels Induced by β-Lactoglobulin. Soft Matter.

[71] Ozel B., Uguz S.S., Kilercioglu M., Grunin L., Oztop M.H. (2017). Effect of Different Polysaccharides on Swelling of Composite Whey Protein Hydrogels: A Low Field (LF) NMR Relaxometry Study. J. Food Process. Eng..

[72] Ge S., Li M., Ji N., Liu J., Mu H., Xiong L., Sun Q. (2018). Preparation of a Strong Gelatin-Short Linear Glucan Nanocomposite Hydrogel by an in Situ Self-Assembly Process. J. Agric. Food Chem..

[73] Gong J., Wang L., Wu J., Yuan Y., Mu R.J., Du Y., Wu C., Pang J. (2019). The Rheological and Physicochemical Properties of a Novel Thermosensitive Hydrogel Based on Konjac Glucomannan/Gum Tragacanth. LWT.

[74] Zhang J., Wang G., Liang Q., Cai W., Zhang Q. (2019). Rheological and Microstructural Properties of Gelatin B/Tara Gum Hydrogels: Effect of Protein/Polysaccharide Ratio, PH and Salt Addition. LWT.

[75] Matalanis A., McClements D.J. (2012). Factors Influencing the Formation and Stability of Filled Hydrogel Particles Fabricated by Protein/Polysaccharide Phase Separation and Enzymatic Cross-Linking. Food Biophys..

[76] Souza Almeida F., Kawazoe Sato A.C. (2019). Structure of Gellan Gum–Hydrolyzed Collagen Particles: Effect of Starch Addition and Coating Layer. Food Res. Int..

[77] Chen Y., Song H., Huang K., Guan X. (2021). Novel Porous Starch/Alginate Hydrogels for Controlled Insulin Release with Dual Response to PH and Amylase. Food Funct..

[78] Yang C., Zhang Z., Liu L., Li Y., Dong X., Chen W. (2022). Fabrication of Soy Protein Isolate/κ-Carrageenan Hydrogels for Release Control of Hydrophilic Compounds: Flax Lignans. Int. J. Biol. Macromol..

[79] Auriemma G., Russo P., del Gaudio P., García-González C.A., Landín M., Aquino R.P. (2020). Technologies and Formulation Design of Polysaccharide-Based Hydrogels for Drug Delivery. Molecules.

[80] Vigata M., Meinert C., Hutmacher D.W., Bock N. (2020). Hydrogels as Drug Delivery Systems: A Review of Current Characterization and Evaluation Techniques. Pharmaceutics.

[81] Bordbar-Khiabani A., Gasik M. (2022). Smart Hydrogels for Advanced Drug Delivery Systems. Int. J. Mol. Sci..

[82] El-Mekawy R.E., Elhady H.A., Al-Shareef H.F. (2021). Highly Stretchable, Smooth, and Biodegradable Hydrogel Films Based on Chitosan as Safety Food Packaging. Polym. Polym. Compos..

[83] Deng J., Zhu E.Q., Xu G.F., Naik N., Murugadoss V., Ma M.G., Guo Z., Shi Z.J. (2022). Overview of Renewable Polysaccharide-Based Composites for Biodegradable Food Packaging Applications. Green Chemistry.

[84] Darabi M.A., Khosrozadeh A., Mbeleck R., Liu Y., Chang Q., Jiang J., Cai J., Wang Q., Luo G., Xing M. (2017). Skin-Inspired Multifunctional Autonomic-Intrinsic Conductive Self-Healing Hydrogels with Pressure Sensitivity, Stretchability, and 3D Printability. Adv. Mater..

[85] Talebian S., Mehrali M., Taebnia N., Pennisi C.P., Kadumudi F.B., Foroughi J., Hasany M., Nikkhah M., Akbari M., Orive G. (2019). Self-Healing Hydrogels: The Next Paradigm Shift in Tissue Engineering?. Adv. Sci..

[86] Guo P., Liang J., Li Y., Lu X., Fu H., Jing H., Guan S., Han D., Niu L. (2019). High-Strength and PH-Responsive Self-Healing Polyvinyl Alcohol/Poly 6-Acrylamidohexanoic Acid Hydrogel Based on Dual Physically Cross-Linked Network. Colloids Surf. A Phys. Eng. Asp..

[87] Mauro M. (2018). Dynamic Metal–Ligand Bonds as Scaffolds for Autonomously Healing Multi-Responsive Materials. Eur. J. Inorg. Chem..

[88] Jing Y., Quan C., Liu B., Jiang Q., Zhang C. (2016). A Mini Review on the Functional Biomaterials Based on Poly(Lactic Acid) Stereocomplex. Polym. Rev..

[89] Han Y., Tan J., Wang D., Xu K., An H. (2019). Novel Approach to Promote the Hydrophobic Association: Introduction of Short Alkyl Chains into Hydrophobically Associating Polyelectrolytes. J. Appl. Polym. Sci..

[90] Su D., Yao M., Liu J., Zhong Y., Chen X., Shao Z. (2017). Enhancing Mechanical Properties of Silk Fibroin Hydrogel through Restricting the Growth of β-Sheet Domains. ACS Appl. Mater. Interfaces.

[91] Choi S., Kwon T., Coskun A., Choi J.W. (2017). Highly Elastic Binders Integrating Polyrotaxanes for Silicon Microparticle Anodes in Lithium Ion Batteries. Science.

[92] Fan Z., Cheng P., Liu M., Li D., Liu G., Zhao Y., Ding Z., Chen F., Wang B., Tan X. (2017). Poly(Glutamic Acid) Hydrogels Crosslinked via Native Chemical Ligation. New J. Chem..

[93] Cai W., Xu D., Qian L., Wei J., Xiao C., Qian L., Lu Z., Cui S. (2019). Force-Induced Transition of π–π Stacking in a Single Polystyrene Chain. J. Am. Chem. Soc..

[94] Sadeghi I., Yi H., Asatekin A. (2018). A Method for Manufacturing Membranes with Ultrathin Hydrogel Selective Layers for Protein Purification: Interfacially Initiated Free Radical Polymerization (IIFRP). Chem. Mater..

[95] Liu Y., Cai Z., Sheng L., Ma M., Xu Q., Jin Y. (2019). Structure-Property of Crosslinked Chitosan/Silica Composite Films Modified by Genipin and Glutaraldehyde under Alkaline Conditions. Carbohydr. Polym..

[96] Nezhad-Mokhtari P., Ghorbani M., Roshangar L., Soleimani Rad J. (2019). A Review on the Construction of Hydrogel Scaffolds by Various Chemically Techniques for Tissue Engineering. Eur. Polym. J..

[97] Tsai C.-C., Hong Y.-J., Lee R.J., Cheng N.-C., Yu J. (2019). Enhancement of Human Adipose-Derived Stem Cell Spheroid Differentiation in an *in Situ* Enzyme-Crosslinked Gelatin Hydrogel. J. Mater. Chem. B.

[98] Choi Y.R., Kim E.H., Lim S., Choi Y.S. (2018). Efficient Preparation of a Permanent Chitosan/Gelatin Hydrogel Using an Acid-Tolerant Tyrosinase. Biochem. Eng. J..

[99] Chen H., Gan J., Ji A., Song S., Yin L. (2019). Development of Double Network Gels Based on Soy Protein Isolate and Sugar Beet Pectin Induced by Thermal Treatment and Laccase Catalysis. Food Chem..

[100] Lei K., Sun Y., Sun C., Zhu D., Zheng Z., Wang X. (2019). Fabrication of a Controlled in Situ Forming Polypeptide Hydrogel with a Good Biological Compatibility and Shapeable Property. ACS Appl. Bio Mater..

[101] Chen F., Chen Q., Zhu L., Tang Z., Li Q., Qin G., Yang J., Zhang Y., Ren B., Zheng J. (2018). General Strategy to Fabricate Strong and Tough Low-Molecular-Weight Gelator-Based Supramolecular Hydrogels with Double Network Structure. Chem. Mater..

[102] Zhang X., Wang J., Jin H., Wang S., Song W. (2018). Bioinspired Supramolecular Lubricating Hydrogel Induced by Shear Force. J. Am. Chem. Soc..

[103] Han J., Cui Y., Han X., Liang C., Liu W., Luo D., Yang D. (2020). Super-Soft DNA/Dopamine-Grafted-Dextran Hydrogel as Dynamic Wire for Electric Circuits Switched by a Microbial Metabolism Process. Adv. Sci..

[104] Munialo C.D., Euston S.R., de Jongh H.H.J. (2018). Protein Gels. Proteins in Food Processing: Second Edition.

[105] Zhang Y.S., Khademhosseini A. (2017). Advances in Engineering Hydrogels. Science.

[106] Liu Y., Fan Y., Wu X., Lu Y., Yi J. (2020). Colloidal Characteristics, Emulsifying Activities, and Interfacial Properties of α-Lactalbumin–Chitosan Electrostatic Complexes: Effects of Mass Ratio and PH. Food Funct..

[107] Sciurti E., Primavera R., di Mascolo D., Rizzo A., Balena A., Padmanabhan S.K., Rizzi F., Decuzzi P., de Vittorio M. (2020). Ultrasound-Induced Deformation of PLGA-MicroPlates for on-Command Drug Release. Microelectron. Eng..

[108] Park H.R., Rho S.J., Kim Y.R. (2019). Solubility, Stability, and Bioaccessibility Improvement of Curcumin Encapsulated Using 4-α-Glucanotransferase-Modified Rice Starch with Reversible PH-Induced Aggregation Property. Food Hydrocoll..

[109] Klost M., Brzeski C., Drusch S. (2020). Effect of Protein Aggregation on Rheological Properties of Pea Protein Gels. Food Hydrocoll..

[110] Zhan X., Dai L., Zhang L., Gao Y. (2020). Entrapment of Curcumin in Whey Protein Isolate and Zein Composite Nanoparticles Using PH-Driven Method. Food Hydrocoll..

[111] Wang X.-Y., Wang J., Rousseau D., Tang C.-H. (2023). Fabrication of Chitosan Colloidal Gels via PH-Mediated Self-Association. Food Hydrocoll..

[112] Talló K., Pons R., González C., López O. (2021). Monitoring the Formation of a Colloidal Lipid Gel at the Nanoscale: Vesicle Aggregation Driven by a Temperature-Induced Mechanism. J. Mater. Chem. B.

[113] Liu K., Li Q.M., Pan L.H., Qian X.P., Zhang H.L., Zha X.Q., Luo J.P. (2017). The Effects of Lotus Root Amylopectin on the Formation of Whey Protein Isolate Gels. Carbohydr. Polym..

[114] Fu H., Li J., Yang X., Swallah M.S., Gong H., Ji L., Meng X., Lyu B., Yu H. (2023). The Heated-Induced Gelation of Soy Protein Isolate at Subunit Level: Exploring the Impacts of α and α′ Subunits on SPI Gelation Based on Natural Hybrid Breeding Varieties. Food Hydrocoll..

[115] Coughlin M.L., Liberman L., Ertem S.P., Edmund J., Bates F.S., Lodge T.P. (2021). Methyl Cellulose Solutions and Gels: Fibril Formation and Gelation Properties. Prog. Polym. Sci..

[116] Wu M., Li R., Liao Q., Wang P., Zhang H. (2023). Thermo-Responsive Behavior and Gelation of Curdlan Alkyl-Ethers Prepared by Homogeneous Reaction. Carbohydr. Polym..

[117] Liu Z., Ren X., Cheng Y., Zhao G., Zhou Y. (2021). Gelation Mechanism of Alkali Induced Heat-Set Konjac Glucomannan Gel. Trends Food Sci. Technol..

[118] Zhao X., Li D., Wang L.J., Wang Y. (2022). Rheological Properties and Microstructure of a Novel Starch-Based Emulsion Gel Produced by One-Step Emulsion Gelation: Effect of Oil Content. Carbohydr. Polym..

[119] Wu C., Hua Y., Chen Y., Kong X., Zhang C. (2017). Effect of Temperature, Ionic Strength and 11S Ratio on the Rheological Properties of Heat-Induced Soy Protein Gels in Relation to Network Proteins Content and Aggregates Size. Food Hydrocoll..

[120] Sriprablom J., Luangpituksa P., Wongkongkatep J., Pongtharangkul T., Suphantharika M. (2019). Influence of PH and Ionic Strength on the Physical and Rheological Properties and Stability of Whey Protein Stabilized o/w Emulsions Containing Xanthan Gum. J. Food Eng..

[121] Zhou F.F., Pan M.K., Liu Y., Guo N., Zhang Q., Wang J.H. (2020). Effects of Na^+^ on the Cold Gelation between a Low-Methoxyl Pectin Extracted from Premna Microphylla Turcz and Soy Protein Isolate. Food Hydrocoll..

[122] Chen S., Zhang Y., Qing J., Han Y., McClements D.J., Gao Y. (2020). Core-Shell Nanoparticles for Co-Encapsulation of Coenzyme Q10 and Piperine: Surface Engineering of Hydrogel Shell around Protein Core. Food Hydrocoll..

[123] Yang J., Li M., Wang Y., Wu H., Zhen T., Xiong L., Sun Q. (2019). Double Cross-Linked Chitosan Composite Films Developed with Oxidized Tannic Acid and Ferric Ions Exhibit High Strength and Excellent Water Resistance. Biomacromolecules.

[124] Gharibzahedi S.M.T., Chronakis I.S. (2018). Crosslinking of Milk Proteins by Microbial Transglutaminase: Utilization in Functional Yogurt Products. Food Chem..

[125] Hoang Thi T.T., Lee Y., le Thi P., Park K.D. (2019). Engineered Horseradish Peroxidase-Catalyzed Hydrogels with High Tissue Adhesiveness for Biomedical Applications. J. Ind. Eng. Chem..

[126] Duquette D., Dumont M.-J. (2019). Comparative Studies of Chemical Crosslinking Reactions and Applications of Bio-Based Hydrogels. Polym. Bull..

[127] Djoullah A., Husson F., Saurel R. (2018). Gelation Behaviors of Denaturated Pea Albumin and Globulin Fractions during Transglutaminase Treatment. Food Hydrocoll..

[128] Zhang M., Yang Y., Acevedo N.C. (2020). Effects of Pre-Heating Soybean Protein Isolate and Transglutaminase Treatments on the Properties of Egg-Soybean Protein Isolate Composite Gels. Food Chem..

[129] Ruzengwe F.M., Amonsou E.O., Kudanga T. (2020). Transglutaminase-Mediated Crosslinking of Bambara Groundnut Protein Hydrogels: Implications on Rheological, Textural and Microstructural Properties. Food Res. Int..

[130] Chen H., Wu D., Ma W., Wu C., Liu J., Du M. (2022). Strong Fish Gelatin Hydrogels Double Crosslinked by Transglutaminase and Carrageenan. Food Chem..

[131] Li Y., Li W., Bao W., Liu B., Li D., Jiang Y., Wei W., Ren F. (2017). Bioinspired Peptosomes with Programmed Stimuli-Responses for Sequential Drug Release and High-Performance Anticancer Therapy. Nanoscale.

[132] Joshi N., Suman K., Joshi Y.M. (2020). Rheological Behavior of Aqueous Poly(Vinyl Alcohol) Solution during a Freeze–Thaw Gelation Process. Macromolecules.

[133] Xu T., Wu J., Zhao L. (2020). Preparation of Grain β-Glucan Gel and Characteristics of Its Slow-Release. J. Food Nutr. Res..

[134] Lewis L., Hatzikiriakos S.G., Hamad W.Y., Maclachlan M.J. (2019). Freeze-Thaw Gelation of Cellulose Nanocrystals. ACS Macro Lett..

[135] Cao C., Li Y. (2020). Highly Stretchable Calcium Ion/Polyacrylic Acid Hydrogel Prepared by Freezing–Thawing. J. Mater. Sci..

[136] Shang L., Wu C., Wang S., Wei X., Li B., Li J. (2021). The Influence of Amylose and Amylopectin on Water Retention Capacity and Texture Properties of Frozen-Thawed Konjac Glucomannan Gel. Food Hydrocoll..

[137] Meng X., Bai Y., Gao J., Li X., Chen H. (2017). Effects of High Hydrostatic Pressure on the Structure and Potential Allergenicity of the Major Allergen Bovine β-Lactoglobulin. Food Chem..

[138] Peyrano F., de Lamballerie M., Avanza M.V., Speroni F. (2021). Gelation of Cowpea Proteins Induced by High Hydrostatic Pressure. Food Hydrocoll..

[139] Guo Z., Li Z., Wang J., Zheng B. (2019). Gelation Properties and Thermal Gelling Mechanism of Golden Threadfin Bream Myosin Containing CaCl_2_ Induced by High Pressure Processing. Food Hydrocoll..

[140] Chen X., Tume R.K., Xiong Y., Xu X., Zhou G., Chen C., Nishiumi T. (2018). Structural Modification of Myofibrillar Proteins by High-Pressure Processing for Functionally Improved, Value-Added, and Healthy Muscle Gelled Foods. Crit. Rev. Food Sci. Nutr..

[141] Luo L., Zhang R., Palmer J., Hemar Y., Yang Z. (2021). Impact of High Hydrostatic Pressure on the Gelation Behavior and Microstructure of Quinoa Protein Isolate Dispersions. ACS Food Sci. Technol..

[142] Liu Y., Chao C., Yu J., Wang S., Wang S., Copeland L. (2020). New Insights into Starch Gelatinization by High Pressure: Comparison with Heat-Gelatinization. Food Chem..

[143] Arnal Á., Royo P., Pataro G., Ferrari G., Ferreira V., López-Sabirón A., Ferreira G. (2018). Implementation of PEF Treatment at Real-Scale Tomatoes Processing Considering LCA Methodology as an Innovation Strategy in the Agri-Food Sector. Sustainability.

[144] Zhu F. (2018). Modifications of Starch by Electric Field Based Techniques. Trends Food Sci. Technol..

[145] Giteru S.G., Oey I., Ali M.A. (2018). Feasibility of Using Pulsed Electric Fields to Modify Biomacromolecules: A Review. Trends Food Sci. Technol..

[146] Soltanzadeh M., Peighambardoust S.H., Gullon P., Hesari J., Gullón B., Alirezalu K., Lorenzo J. (2020). Quality Aspects and Safety of Pulsed Electric Field (PEF) Processing on Dairy Products: A Comprehensive Review. Food Rev. Int..

[147] Jin W., Wang Z., Peng D., Shen W., Zhu Z., Cheng S., Li B., Huang Q. (2020). Effect of Pulsed Electric Field on Assembly Structure of α-Amylase and Pectin Electrostatic Complexes. Food Hydrocoll..

[148] Taha A., Casanova F., Šimonis P., Stankevič V., Gomaa M.A.E., Stirkė A. (2022). Pulsed Electric Field: Fundamentals and Effects on the Structural and Techno-Functional Properties of Dairy and Plant Proteins. Foods.

[149] Nunes L., Tavares G.M. (2019). Thermal Treatments and Emerging Technologies: Impacts on the Structure and Techno-Functional Properties of Milk Proteins. Trends Food Sci. Technol..

[150] Wang Q., Wei R., Hu J., Luan Y., Liu R., Ge Q., Yu H., Wu M. (2022). Moderate Pulsed Electric Field-Induced Structural Unfolding Ameliorated the Gelling Properties of Porcine Muscle Myofibrillar Protein. Innov. Food Sci. Emerg. Technol..

[151] Dong M., Tian H., Xu Y., Han M., Xu X. (2021). Effects of Pulsed Electric Fields on the Conformation and Gelation Properties of Myofibrillar Proteins Isolated from Pale, Soft, Exudative (PSE)-like Chicken Breast Meat: A Molecular Dynamics Study. Food Chem..

[152] Zhu Q.L., Dai C.F., Wagner D., Daab M., Hong W., Breu J., Zheng Q., Wu Z.L. (2020). Distributed Electric Field Induces Orientations of Nanosheets to Prepare Hydrogels with Elaborate Ordered Structures and Programmed Deformations. Adv. Mater..

[153] Giteru S.G., Azam Ali M., Oey I. (2021). Elucidating the PH Influence on Pulsed Electric Fields-Induced Self-Assembly of Chitosan-Zein-Poly(Vinyl Alcohol)-Polyethylene Glycol Nanostructured Composites. J. Colloid Interface Sci..

[154] Brindle L.P., Krochta J.M. (2008). Physical Properties of Whey Protein-Hydroxypropylmethylcellulose Blend Edible Films. J. Food Sci..

[155] Yoo S., Krochta J.M. (2011). Whey Protein-Polysaccharide Blended Edible Film Formation and Barrier, Tensile, Thermal and Transparency Properties. J. Sci. Food Agric..

[156] Wang L.F., Rhim J.W. (2015). Preparation and Application of Agar/Alginate/Collagen Ternary Blend Functional Food Packaging Films. Int. J. Biol. Macromol..

[157] Zhang H., Zhang F., Yuan R. (2020). Applications of Natural Polymer-Based Hydrogels in the Food Industry. Hydrogels Based on Natural Polymers.

[158] De Souza Paglarini C., Martini S., Pollonio M.A.R. (2019). Using Emulsion Gels Made with Sonicated Soy Protein Isolate Dispersions to Replace Fat in Frankfurters. LWT.

[159] Domínguez R., Munekata P.E., Pateiro M., López-Fernández O., Lorenzo J.M. (2021). Immobilization of Oils Using Hydrogels as Strategy to Replace Animal Fats and Improve the Healthiness of Meat Products. Curr. Opin. Food Sci..

[160] Barragán-Martínez L.P., Román-Guerrero A., Vernon-Carter E.J., Alvarez-Ramirez J. (2022). Impact of Fat Replacement by a Hybrid Gel (Canola Oil/Candelilla Wax Oleogel and Gelatinized Corn Starch Hydrogel) on Dough Viscoelasticity, Color, Texture, Structure, and Starch Digestibility of Sugar-Snap Cookies. Int. J. Gastron. Food Sci..

[161] Bandyopadhyay S., Saha N., Brodnjak U.V., Saha P. (2018). Bacterial Cellulose Based Greener Packaging Material: A Bioadhesive Polymeric Film. Mater. Res. Express.

[162] Batista R.A., Espitia P.J.P., Quintans J.D.S.S., Freitas M.M., Cerqueira M.Â., Teixeira J.A., Cardoso J.C. (2019). Hydrogel as an Alternative Structure for Food Packaging Systems. Carbohydr. Polym..

[163] Lu P., Yang Y., Liu R., Liu X., Ma J., Wu M., Wang S. (2020). Preparation of Sugarcane Bagasse Nanocellulose Hydrogel as a Colourimetric Freshness Indicator for Intelligent Food Packaging. Carbohydr. Polym..

[164] Safitri E.A., Mahendra I.P., Putra A.E., Ghifari M.A., Yanti D.D., Yusnaidar Y., Ariwahjoedi B., Mendez J.A. (2021). Multicolor PEGDA/LCNF Hydrogel in the Presence of Red Cabbage Anthocyanin Extract. Gels.

[165] Sutthasupa S., Padungkit C., Suriyong S. (2021). Colorimetric Ammonia (NH_3_) Sensor Based on an Alginate-Methylcellulose Blend Hydrogel and the Potential Opportunity for the Development of a Minced Pork Spoilage Indicator. Food Chem..

[166] Tuorila H., Hartmann C. (2020). Consumer Responses to Novel and Unfamiliar Foods. Curr. Opin. Food Sci..

[167] McClements D.J., Grossmann L. (2021). A Brief Review of the Science behind the Design of Healthy and Sustainable Plant-Based Foods. NPJ Sci. Food.

[168] Ozel B., Cikrikci S., Aydin O., Oztop M.H. (2017). Polysaccharide Blended Whey Protein Isolate-(WPI) Hydrogels: A Physicochemical and Controlled Release Study. Food Hydrocoll..

[169] Valencia G.A., Zare E.N., Makvandi P., Gutiérrez T.J. (2019). Self-Assembled Carbohydrate Polymers for Food Applications: A Review. Compr. Rev. Food Sci. Food Saf..

[170] Tan Y., McClements D.J. (2021). Plant-Based Colloidal Delivery Systems for Bioactives. Molecules.

[171] Wijaya W., Patel A.R., Setiowati A.D., van der Meeren P. (2017). Functional Colloids from Proteins and Polysaccharides for Food Applications. Trends Food Sci. Technol..

[172] Farjami T., Madadlou A. (2019). An Overview on Preparation of Emulsion-Filled Gels and Emulsion Particulate Gels. Trends Food Sci. Technol..

[173] Liu K., Huang R.L., Zha X.Q., Li Q.M., Pan L.H., Luo J.P. (2020). Encapsulation and Sustained Release of Curcumin by a Composite Hydrogel of Lotus Root Amylopectin and Chitosan. Carbohydr. Polym..

[174] Huang H., Belwal T., Aalim H., Li L., Lin X., Liu S., Ma C., Li Q., Zou Y., Luo Z. (2019). Protein-Polysaccharide Complex Coated W/O/W Emulsion as Secondary Microcapsule for Hydrophilic Arbutin and Hydrophobic Coumaric Acid. Food Chem..

[175] Araiza-Calahorra A., Akhtar M., Sarkar A. (2018). Recent Advances in Emulsion-Based Delivery Approaches for Curcumin: From Encapsulation to Bioaccessibility. Trends Food Sci. Technol..

[176] George D., Maheswari P.U., Sheriffa Begum K.M.M., Arthanareeswaran G. (2019). Biomass-Derived Dialdehyde Cellulose Cross-Linked Chitosan-Based Nanocomposite Hydrogel with Phytosynthesized Zinc Oxide Nanoparticles for Enhanced Curcumin Delivery and Bioactivity. J. Agric. Food Chem..

[177] Zhang C., Wang X., Xiao M., Ma J., Qu Y., Zou L., Zhang J. (2022). Nano-in-Micro Alginate/Chitosan Hydrogel via Electrospray Technology for Orally Curcumin Delivery to Effectively Alleviate Ulcerative Colitis. Mater. Des..

[178] Kour P., Afzal S., Gani A., Zargar M.I., Nabi Tak U., Rashid S., Dar A.A. (2022). Effect of Nanoemulsion-Loaded Hybrid Biopolymeric Hydrogel Beads on the Release Kinetics, Antioxidant Potential and Antibacterial Activity of Encapsulated Curcumin. Food Chem..

[179] Wani T.A., Shah A.G., Wani S.M., Wani I.A., Masoodi F.A., Nissar N., Shagoo M.A. (2016). Suitability of Different Food Grade Materials for the Encapsulation of Some Functional Foods Well Reported for Their Advantages and Susceptibility. Crit. Rev. Food Sci. Nutr..

[180] Wang L., Zhou N., Zheng S., Pang J. (2022). Formation of Composite Hydrogel of Carboxymethyl Konjac Glucomannan/Gelatin for Sustained Release of EGCG. Food Sci. Hum. Wellness.

[181] Yu X., Li J., Yang M., Chen C., Munir S., You J., Yin T., Liu R., Xiong S., Hu Y. (2021). Role of Epigallocatechin Gallate in Collagen Hydrogels Modification Based on Physicochemical Characterization and Molecular Docking. Food Chem..

[182] Lee G.M., Kim S., Kim E.M., Kim E., Lee S., Lee E., Park H.H., Shin H. (2022). Free Radical-Scavenging Composite Gelatin Methacryloyl Hydrogels for Cell Encapsulation. Acta Biomater..

[183] Wu H., Bu N., Chen J., Chen Y., Sun R., Wu C., Pang J. (2022). Construction of Konjac Glucomannan/Oxidized Hyaluronic Acid Hydrogels for Controlled Drug Release. Polymers.

[184] Xu Z., Shan G., Hao N., Li L., Lan T., Dong Y., Wen J., Tian R., Zhang Y., Jiang L. (2022). Structure Remodeling of Soy Protein-Derived Amyloid Fibrils Mediated by Epigallocatechin-3-Gallate. Biomaterials.

[185] Rauf A., Imran M., Suleria H.A.R., Ahmad B., Peters D.G., Mubarak M.S. (2017). A Comprehensive Review of the Health Perspectives of Resveratrol. Food Funct..

[186] Fan Y., Zeng X., Yi J., Zhang Y. (2020). Fabrication of Pea Protein Nanoparticles with Calcium-Induced Cross-Linking for the Stabilization and Delivery of Antioxidative Resveratrol. Int. J. Biol. Macromol..

[187] Miao L., Daozhou L., Ying C., Qibing M., Siyuan Z. (2021). A Resveratrol-Loaded Nanostructured Lipid Carrier Hydrogel to Enhance the Anti-UV Irradiation and Anti-Oxidant Efficacy. Colloids Surf. B Biointerfaces.

[188] Wu B., Li Y., Li Y., Li H., Li L., Xia Q. (2022). Encapsulation of Resveratrol-Loaded Pickering Emulsions in Alginate/Pectin Hydrogel Beads: Improved Stability and Modification of Digestive Behavior in the Gastrointestinal Tract. Int. J. Bio.l Macromol..

[189] Rawdkuen S., Faseha A., Benjakul S., Kaewprachu P. (2020). Application of Anthocyanin as a Color Indicator in Gelatin Films. Food Biosci..

[190] Jin W., Xiang L., Peng D., Liu G., He J., Cheng S., Li B., Huang Q. (2020). Study on the Coupling Progress of Thermo-Induced Anthocyanins Degradation and Polysaccharides Gelation. Food Hydrocoll..

[191] Ćorković I., Pichler A., Buljeta I., Šimunović J., Kopjar M. (2021). Carboxymethylcellulose Hydrogels: Effect of Its Different Amount on Preservation of Tart Cherry Anthocyanins and Polyphenols. Curr. Plant Biol..

[192] Liu L., Zhang D., Song X., Guo M., Wang Z., Geng F., Zhou X., Nie S. (2022). Compound Hydrogels Derived from Gelatin and Gellan Gum Regulates the Release of Anthocyanins in Simulated Digestion. Food Hydrocoll..

[193] Kopjar M., Ćorković I., Buljeta I., Šimunović J., Pichler A. (2022). Fortification of Pectin/Blackberry Hydrogels with Apple Fibers: Effect on Phenolics, Antioxidant Activity and Inhibition of α-Glucosidase. Antioxidants.

[194] Viscusi G., Lamberti E., Gerardi C., Giovinazzo G., Gorrasi G. (2022). Encapsulation of Grape (*Vitis vinifera* L.) Pomace Polyphenols in Soybean Extract-Based Hydrogel Beads as Carriers of Polyphenols and PH-Monitoring Devices. Gels.

[195] Zhai X., Sun Y., Cen S., Wang X., Zhang J., Yang Z., Li Y., Wang X., Zhou C., Arslan M. (2022). Anthocyanins-Encapsulated 3D-Printable Bigels: A Colorimetric and Leaching-Resistant Volatile Amines Sensor for Intelligent Food Packaging. Food Hydrocoll..

[196] Oh W.Y., Ambigaipalan P., Shahidi F. (2019). Preparation of Quercetin Esters and Their Antioxidant Activity. J. Agric. Food Chem..

[197] Liu K., Zha X.-Q., Shen W.-D., Li Q.-M., Pan L.-H., Luo J.-P. (2020). The Hydrogel of Whey Protein Isolate Coated by Lotus Root Amylopectin Enhance the Stability and Bioavailability of Quercetin. Carbohydr. Polym..

[198] Huang J., Wang Q., Chu L., Xia Q. (2020). Liposome-Chitosan Hydrogel Bead Delivery System for the Encapsulation of Linseed Oil and Quercetin: Preparation and in Vitro Characterization Studies. LWT.

[199] Hu M., Liu G., Zhang W., Du X., Qi B., Li Y. (2022). Co-Encapsulation of (–)-Epigallocatechin-3-Gallate and Quercetin in Double Emulsion Hydrogel Beads: Microstructures, Functional Properties, and Digestion Behaviors. Food Chem..

[200] Gupta C., Arora S., Syama M.A., Sharma A. (2017). Preparation of Milk Protein-Vitamin A Complexes and Their Evaluation for Vitamin A Binding Ability. Food Chem..

[201] Rana S., Arora S., Gupta C., Bodemala H., Kapila S. (2021). Evaluation of In-Vivo Model for Vitamin A Bioavailability from Vitamin A Loaded Caseinate Complex. Food Biosci..

[202] Kaur K., Jindal R., Jindal D. (2020). Controlled Release of Vitamin B1 and Evaluation of Biodegradation Studies of Chitosan and Gelatin Based Hydrogels. Int. J. Biol. Macromol..

[203] Kundu D., Banerjee T. (2019). Carboxymethyl Cellulose-Xylan Hydrogel: Synthesis, Characterization, and in Vitro Release of Vitamin B 12. ACS Omega.

[204] Eid M., Sobhy R., Zhou P., Wei X., Wu D., Li B. (2020). β-Cyclodextrin- Soy Soluble Polysaccharide Based Core-Shell Bionanocomposites Hydrogel for Vitamin E Swelling Controlled Delivery. Food Hydrocoll..

[205] Martinez R.M., Magalhães W.V., da Silva Sufi B., Padovani G., Nazato L.I.S., Velasco M.V.R., da Silva Lannes S.C., Baby A.R. (2021). Vitamin E-Loaded Bigels and Emulsions: Physicochemical Characterization and Potential Biological Application. Colloids Surf. B Biointerfaces.

[206] Mir T.A., Ali A., Mazumdar N. (2022). Glycerol-Crosslinked Guar Gum Monoaldehyde Based Superabsorbent Hydrogels for Vitamin B6 (Pyridoxine Hydrochloride) Delivery. Polym. Bull..

[207] McClements D.J. (2017). Designing Biopolymer Microgels to Encapsulate, Protect and Deliver Bioactive Components: Physicochemical Aspects. Adv. Colloid Interface Sci..

[208] McClements D.J. (2018). Encapsulation, Protection, and Delivery of Bioactive Proteins and Peptides Using Nanoparticle and Microparticle Systems: A Review. Adv. Colloid Interface Sci..

[209] Wei Z., Volkova E., Blatchley M.R., Gerecht S. (2019). Hydrogel Vehicles for Sequential Delivery of Protein Drugs to Promote Vascular Regeneration. Adv. Drug Deliv. Rev..

[210] Bhat Z.F., Morton J.D., Bekhit A.E.A., Kumar S., Bhat H.F. (2021). Effect of Processing Technologies on the Digestibility of Egg Proteins. Compr. Rev. Food Sci. Food Saf..

[211] Punia S. (2020). Barley Starch: Structure, Properties and in Vitro Digestibility—A Review. Int. J. Biol. Macromol..

[212] Wealleans A.L., Walsh M.C., Romero L.F., Ravindran V. (2017). Comparative Effects of Two Multi-Enzyme Combinations and a Bacillus Probiotic on Growth Performance, Digestibility of Energy and Nutrients, Disappearance of Non-Starch Polysaccharides, and Gut Microflora in Broiler Chickens. Poult. Sci..

[213] Florowska A., Hilal A., Florowski T., Brandelli A. (2022). Prebiotics and Synbiotics. Probiotics.

[214] Mahinroosta M., Jomeh Farsangi Z., Allahverdi A., Shakoori Z. (2018). Hydrogels as Intelligent Materials: A Brief Review of Synthesis, Properties and Applications. Mater. Today Chem..

[215] Li Z., Zhang L., Mao C., Song Z., Li X., Liu C. (2021). Preparation and Characterization of Konjac Glucomannan and Gum Arabic Composite Gel. Int. J. Biol. Macromol..

[216] McClements D.J. (2017). Recent Progress in Hydrogel Delivery Systems for Improving Nutraceutical Bioavailability. Food Hydrocoll..

[217] Mezzenga R., Fischer P. (2013). The Self-Assembly, Aggregation and Phase Transitions of Food Protein Systems in One, Two and Three Dimensions. Rep. Prog. Phys..

[218] Tulain U.R., Ahmad M., Rashid A., Malik M.Z., Iqbal F.M. (2018). Fabrication of PH-Responsive Hydrogel and Its In Vitro and In Vivo Evaluation. Adv. Polym. Technol..

[219] Xie A.-J., Yin H.-S., Liu H.-M., Zhu C.-Y., Yang Y.-J. (2018). Chinese Quince Seed Gum and Poly (N,N-Diethylacryl Amide-Co-Methacrylic Acid) Based PH-Sensitive Hydrogel for Use in Drug Delivery. Carbohydr. Polym..

[220] Sarıyer S., Duranoğlu D., Doğan Ö., Küçük İ. (2020). PH-Responsive Double Network Alginate/Kappa-Carrageenan Hydrogel Beads for Controlled Protein Release: Effect of PH and Crosslinking Agent. J. Drug Deliv. Sci. Technol..

[221] Shaghaleh H., Hamoud Y.A., Xu X., Liu H., Wang S., Sheteiwy M., Dong F., Guo L., Qian Y., Li P. (2021). Thermo-/PH-Responsive Preservative Delivery Based on TEMPO Cellulose Nanofiber/Cationic Copolymer Hydrogel Film in Fruit Packaging. Int. J. Biol. Macromol..

[222] Baus R.A., Zahir-Jouzdani F., Dünnhaupt S., Atyabi F., Bernkop-Schnürch A. (2019). Mucoadhesive Hydrogels for Buccal Drug Delivery: In Vitro-in Vivo Correlation Study. Eur. J. Pharm. Biopharm..

[223] Wang C.-Y., Sun M., Fan Z., Du J.-Z. (2022). Intestine Enzyme-Responsive Polysaccharide-Based Hydrogel to Open Epithelial Tight Junctions for Oral Delivery of Imatinib against Colon Cancer. Chin. J. Polym. Sci..

[224] Zhao H., Li Y. (2020). A Novel PH/Temperature-Responsive Hydrogel Based on Tremella Polysaccharide and Poly(N-Isopropylacrylamide). Colloids Surf. A Physicochem. Eng. Asp..

[225] Liao J., Huang H. (2020). Smart PH/Magnetic Sensitive *Hericium erinaceus* Residue Carboxymethyl Chitin/Fe_3_O_4_ Nanocomposite Hydrogels with Adjustable Characteristics. Carbohydr. Polym..

